# Abnormal hippocampal structure and function in juvenile myoclonic epilepsy and unaffected siblings

**DOI:** 10.1093/brain/awz215

**Published:** 2019-07-31

**Authors:** Lorenzo Caciagli, Britta Wandschneider, Fenglai Xiao, Christian Vollmar, Maria Centeno, Sjoerd B Vos, Karin Trimmel, Meneka K Sidhu, Pamela J Thompson, Gavin P Winston, John S Duncan, Matthias J Koepp

**Affiliations:** 1 Department of Clinical and Experimental Epilepsy, UCL Queen Square Institute of Neurology, Queen Square, London, UK; 2 MRI Unit, Epilepsy Society, Chalfont St Peter, Buckinghamshire, UK; 3 Department of Neurology, West China Hospital of Sichuan University, Chengdu, Sichuan, China; 4 Department of Neurology, Ludwig-Maximilians-Universität, Marchioninistrasse 15, Munich, Germany; 5 Wellcome/EPSRC Centre for Interventional and Surgical Sciences, University College London, London, UK; 6 Centre for Medical Image Computing, University College London, London, UK; 7 Department of Neurology, Medical University of Vienna, Vienna, Austria; 8 Department of Medicine, Division of Neurology, Queen’s University, Kingston, Ontario, Canada

**Keywords:** juvenile myoclonic epilepsy, magnetic resonance imaging, endophenotype, hippocampal malrotation, memory

## Abstract

Juvenile myoclonic epilepsy is the most common genetic generalized epilepsy syndrome, characterized by a complex polygenetic aetiology. Structural and functional MRI studies demonstrated mesial or lateral frontal cortical derangements and impaired fronto-cortico-subcortical connectivity in patients and their unaffected siblings. The presence of hippocampal abnormalities and associated memory deficits is controversial, and functional MRI studies in juvenile myoclonic epilepsy have not tested hippocampal activation. In this observational study, we implemented multi-modal MRI and neuropsychological data to investigate hippocampal structure and function in 37 patients with juvenile myoclonic epilepsy, 16 unaffected siblings and 20 healthy controls, comparable for age, gender, handedness and hemispheric dominance as assessed with language laterality indices. Automated hippocampal volumetry was complemented by validated qualitative and quantitative morphological criteria to detect hippocampal malrotation, assumed to represent a neurodevelopmental marker. Neuropsychological measures of verbal and visuo-spatial learning and an event-related verbal and visual memory functional MRI paradigm addressed mesiotemporal function. We detected a reduction of mean left hippocampal volume in patients and their siblings compared with controls (*P < *0.01). Unilateral or bilateral hippocampal malrotation was identified in 51% of patients and 50% of siblings, against 15% of controls (*P < *0.05). For bilateral hippocampi, quantitative markers of verticalization had significantly larger values in patients and siblings compared with controls (*P < *0.05). In the patient subgroup, there was no relationship between structural measures and age at disease onset or degree of seizure control. No overt impairment of verbal and visual memory was identified with neuropsychological tests. Functional mapping highlighted atypical patterns of hippocampal activation, pointing to abnormal recruitment during verbal encoding in patients and their siblings [*P < *0.05, familywise error (FWE)-corrected]. Subgroup analyses indicated distinct profiles of hypoactivation along the hippocampal long axis in juvenile myoclonic epilepsy patients with and without malrotation; patients with malrotation also exhibited reduced frontal recruitment for verbal memory, and more pronounced left posterior hippocampal involvement for visual memory. Linear models across the entire study cohort indicated significant associations between morphological markers of hippocampal positioning and hippocampal activation for verbal items (all *P < *0.05, FWE-corrected). We demonstrate abnormalities of hippocampal volume, shape and positioning in patients with juvenile myoclonic epilepsy and their siblings, which are associated with reorganization of function and imply an underlying neurodevelopmental mechanism with expression during the prenatal stage. Co-segregation of abnormal hippocampal morphology in patients and their siblings is suggestive of a genetic imaging phenotype, independent of disease activity, and can be construed as a novel endophenotype of juvenile myoclonic epilepsy.

## Teaser

Using multi-modal MRI, Caciagli *et al.* document abnormalities of hippocampal volume, shape and positioning in patients with juvenile myoclonic epilepsy and their unaffected siblings, which are associated with altered memory-associated functional activations. These findings represent novel genetic imaging phenotypes, and imply a neurodevelopmental mechanism with expression during the prenatal stage.

## Introduction

Juvenile myoclonic epilepsy (JME) is a highly prevalent genetic generalized epilepsy syndrome, accounting for up to 10% of all epilepsies (Camfield *et al.*, 2013; Scheffer *et al.*, 2017). Complex polygenetic transmission mechanisms contribute to the aetiology of JME (Delgado-Escueta *et al.*, 2013). Clinical genetic investigations document high levels of syndrome concordance in monozygotic twins (Berkovic *et al.*, 1998; Corey *et al.*, 2011) and first-degree relatives of index patients (Marini *et al.*, 2004), further supporting high heritability.

Neuropsychological evaluations in JME have shown frontal lobe dysfunction, with impaired performance during working memory, prospective memory, decision-making and other executive tasks (Sonmez *et al.*, 2004; Wandschneider *et al.*, 2012; Zamarian *et al.*, 2013; Wolf *et al.*, 2015), and specific personality traits (Plattner *et al.*, 2007; de Araujo Filho and Yacubian, 2013). Abnormal fronto-cortico-thalamic connections (Pulsipher *et al.*, 2009; O’Muircheartaigh *et al.*, 2011, 2012) and augmented structural and functional connectivity between motor areas and prefrontal cognitive networks (Vollmar *et al.*, 2011, 2012) have been interpreted as substrates of both ictogenesis and cognitive dysfunction (Koepp, 2005; Wandschneider *et al.*, 2012).

While most cognitive and imaging investigations have focused on the frontal lobes, structure and function of temporal lobe areas, particularly the hippocampus, are less well characterized. Previous work detected left (Tae *et al.*, 2006), right (Lin *et al.*, 2013) or bilateral hippocampal volume loss (Kim *et al.*, 2015), abnormal lateral temporal cortical morphology (Ronan *et al.*, 2012) and disrupted maturational trajectories of temporo-parietal association cortices (Lin *et al.*, 2014). Other than atrophy, subtle abnormalities include atypical appearance and positioning of the hippocampal formation, with rounded or pyramidal shape and abnormal collateral or occipito-temporal sulcal morphometry, an entity known as hippocampal malrotation (HIMAL) (Bernasconi *et al.*, 2005; Tsai *et al.*, 2016). HIMAL is more common in individuals with various focal epilepsy syndromes than in the general population (Bernasconi *et al.*, 2005), and is ascribed to incomplete inversion of mesiotemporal structures during gestation, representing a neurodevelopmental marker (Bajic *et al.*, 2010). Its prevalence in a syndrome underpinned by abnormalities of neurodevelopment, such as JME, and its potential functional consequences remain to be established.

As for temporal lobe functions, some studies reported unaffected verbal and non-verbal memory in JME (Roebling *et al.*, 2009; Wandschneider *et al.*, 2010), though memory deficits encompassing verbal and visuo-spatial domains were detected by others (Sonmez *et al.*, 2004; Pascalicchio *et al.*, 2007; Giorgi *et al.*, 2016), and further documented by a recent meta-analysis (Loughman *et al.*, 2014). Functional MRI paradigms highlight the neural correlates of episodic memory encoding (Kim, 2011; Sidhu *et al.*, 2013; Voets *et al.*, 2014), with event-related designs specifically addressing subsequent memory effects, and indicating a pivotal role of mesiotemporal structures (Bonelli *et al.*, 2010; Sidhu *et al.*, 2013). To date, no study has addressed the imaging correlates of mesiotemporal function in JME.

This study was designed to investigate structure and function of the mesiotemporal lobes in JME. We used structural MRI to provide quantitative measures of hippocampal volumes. In view of the neurodevelopmental underpinnings of JME, we tested for markers of atypical hippocampal morphology pointing to HIMAL. Neuropsychological tests were implemented to address verbal and visuo-spatial learning, while an event-related analysis of a memory encoding functional MRI paradigm, including both verbal and visual items, was used to explore mesiotemporal activation. Given the polygenetic background and recent evidence of overlapping imaging and neurobehavioural traits in JME patients and unaffected relatives (Wandschneider *et al.*, 2014; Iqbal *et al.*, 2015), the above measures were also obtained for unaffected JME siblings. This provides a framework to address trait heritability, and detect JME-related endophenotypes, i.e. heritable traits associated with the disease at the population level, co-segregating in families with affected members, which may closely relate to disease pathophysiological mechanisms (Gottesman and Gould, 2003).

## Materials and methods

### Participants

In this observational study, we consecutively recruited 37 patients with JME, 16 unaffected siblings of 11 index patients, and 20 healthy control subjects. Eighteen of 32 patients (56%) and 11 of 16 siblings (69%) reported a family history of epilepsy (beyond the JME index patient, for siblings), while reliable anamnestic information was not available for five patients. All controls had no family history of epilepsy. Patients with JME were recruited from outpatient clinics at the National Hospital for Neurology and Neurosurgery and Epilepsy Society. Siblings were recruited through index patients. Controls were recruited from the local community (North-West London and Chalfont St Peter, Buckinghamshire, UK). All patients had previously undergone a structural MRI scan as part of diagnostic investigations. None of the siblings and controls had received an MRI scan prior to participating in this study. Demographic and clinical details are reported in Table 1. The groups were comparable for age, gender, handedness and anxiety/depression scores. Language laterality indices, derived from individual verbal fluency functional MRI with the bootstrap method of the Statistical Parametric Mapping (SPM) LI toolbox (Wilke and Lidzba, 2007; Centeno *et al.*, 2014) did not differ among groups. All patients had a typical history of JME, with onset of myoclonic and generalized tonic-clonic seizures during adolescence, and absence seizures documented for some (40.5%). At least one routine scalp EEG showed generalized polyspike-wave discharges, and routine brain MRI scans were normal. JME siblings and controls had never experienced unprovoked seizures. EEG investigations could not be performed in these subjects because of ethics restrictions. Recruitment for this study received approval by the London South-East Research Ethics Committee and by the UCL/UCLH Joint Research Office. Written informed consent was obtained from all participants.

**Table 1:** Demographic details, clinical characteristics and neuropsychological test results

	JME	SIB	CTR	Test statistic	*P*-value	*Post hoc P*-value
Age*,* median (IQR)	32.0 (14.0)	41.5 (20.0)	32.5 (7.0)	2.53^#^	0.28	–
Gender*,* F/M	20/17	10/6	13/7	0.75*	0.74	–
Handedness, L/R	2/35	2/14	2/18	1.28*	0.62	–
HADS/A*,* median (IQR)	6 (4)	5 (2)	4 (5)	5.10^#^	0.08	–
HADS/D*,* median (IQR)	2 (4)	1 (2)	1 (2)	4.00^#^	0.14	–
Language LI*,* median (IQR)	0.67 (0.28)	0.68 (0.17)	0.70 (0.34)	0.71^#^	0.70	–
NART IQ*,* median (IQR)	111.0 (13.3)	106.5 (18.8)	109.0 (10.0)	0.87^#^	0.65	–
List Learning (A1–5), median (IQR)	56.0 (16.0)	56.5 (14.8)	56.0 (12.3)	2.02^#^	0.37	–
List Learning (A6)*,* median (IQR)	12.0 (5.0)	12.0 (3.0)	13.0 (2.3)	0.74^#^	0.69	–
Design Learning (A1–5)*,* median (IQR)	38.0 (12.0)	36.5 (10.3)	39.5 (13.0)	1.68^#^	0.43	–
Design Learning (A6)*,* median (IQR)	8.0 (3.0)	9.0 (1.0)	9.0 (2.0)	6.26^#^	0.04	CTR/SIB: 0.12
						CTR/JME: 0.15
						JME/SIB: 1.00
Recognition accuracy: fMRI Task (Words)*,* median (IQR)	80.0 (19.3)	80.0 (22.5)	84.3 (35.7)	0.40^#^	0.82	–
Recognition accuracy: fMRI Task (Faces)*,* median (IQR)	27.1 (17.9)	27.9 (20.7)	37.1 (25.7)	3.68^#^	0.16	–
History of febrile seizures*, n*, (%)	5 (13.5)	0	0	N/A	N/A	–
Age at disease onset*,* years median (IQR)	15.0 (4.0)	N/A	N/A	N/A	N/A	–
Disease duration*,* years, median (IQR)	19.0 (16.0)	N/A	N/A	N/A	N/A	–
Time since last seizure*,* years, median (IQR)	1.1 (4.7)	N/A	N/A	N/A	N/A	–
AEDs at time of scan*,* median (IQR)	2.0 (1.0)	N/A	N/A	N/A	N/A	–
AEDs trialled since disease onset, median (IQR)	3.0 (2.25)	N/A	N/A	N/A	N/A	–

Patients were recruited from outpatient clinics at the National Hospital for Neurology and Neurosurgery and Epilepsy Society. Siblings were recruited through index patients. Controls were recruited from the local community. Handedness was assessed via the Edinburgh Handedness Inventory. HADS/A scores pertain to anxiety-related symptom, HADS/D scores refer to depression symptoms. List/Design Learning and Recall scores are reported as raw, and were not available in seven controls and two JME patients. Pairwise deletion was applied in case of missing data. Details are provided in the main text. All *P*-values are reported as uncorrected for multiple comparisons. *Post hoc P*-values are Bonferroni-corrected for multiple comparisons.

AED = anti-epileptic drug; CTR = controls; fMRI = functional MRI; HADS = Hospital Anxiety and Depression Scale; LI = laterality index; SIB = siblings of patients with JME.

^#^Kruskal-Wallis test, χ^2^ statistic; *Pearson’s χ^2^.

### Imaging data acquisition

T_1_-weighted structural MRI data were acquired for all participants on a 3 T GE Signa-HDx MRI scanner with an 8-channel head coil, using a 3D fast-spoiled gradient-echo (FSPGR) sequence with acquisition perpendicular to the hippocampal long axis, matrix size 256 × 256, isotropic voxel size: 1.1 mm, echo time/repetition time/inversion time: 2.8/7.2/450 ms, flip angle: 20°. Functional MRI data were obtained using a 50-slice gradient echo-planar sequence with axial orientation, 64 × 64 matrix corresponding to in-plane voxel size of 3.75 × 3.75 mm, 2.4 mm slice thickness, 0.1 mm inter-slice gap, echo time/repetition time: 25/2500 ms, flip angle: 70°.

### Structural imaging analysis

#### Hippocampal volumetry

Hippocampal segmentation was carried out on T_1_-weighted images using Hipposeg (https://hipposeg.cs.ucl.ac.uk/), an open-source multi-atlas-based segmentation algorithm, and correction of individual hippocampal volume for total intracranial volume was achieved via linear regression, as detailed previously (Winston *et al.*, 2013). The occurrence of hippocampal shape variants, including HIMAL, may decrease the accuracy of automated segmentation methods (Kim *et al.*, 2012). Hence, all the hippocampal masks were visually verified by one experienced investigator (L.C.), blinded to the subject identity, and corrected if appropriate. Minor corrections (<45 mm^3^) were applied in 47/73 (64%) cases. In one subject, more extensive correction was necessary owing to the erroneous inclusion of the fundus of the collateral sulcus. Intra-rater reliability after mask correction, calculated on a randomly selected data subset (*n* = 30) and based on two sessions 6 months apart, gave an intraclass correlation coefficient (ICC) of 0.95.

#### Qualitative and quantitative assessment of hippocampal malrotation

**Figure 1:**
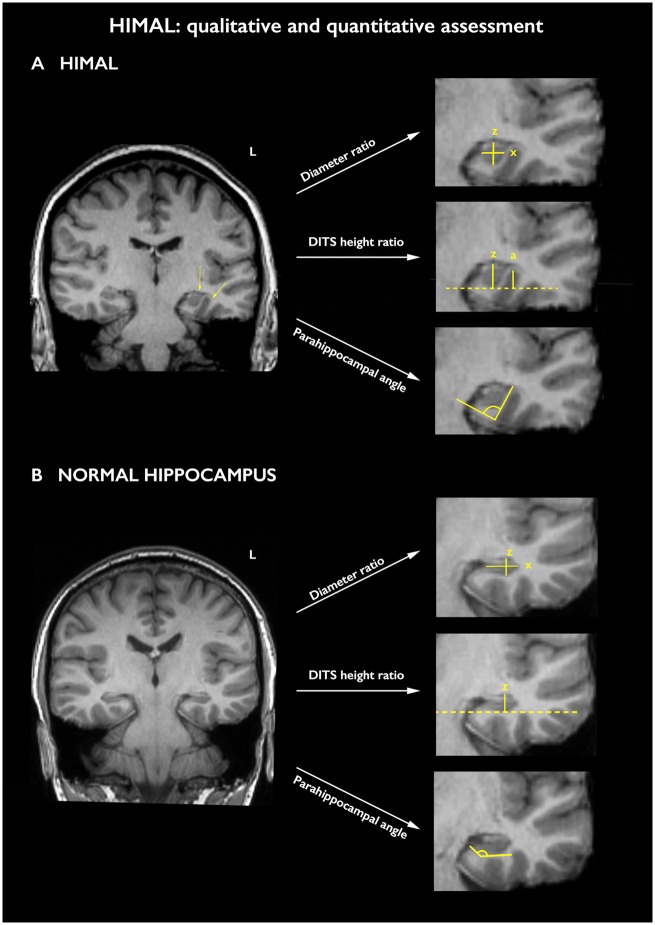
**Qualitative and quantitative assessment of the hippocampus.** (**A**) An example of a left hippocampus with HIMAL, as evidenced by a round hippocampal shape, loss of the lateral hippocampal convexity, the latter implying an enhanced radius of curvature (left-hand arrow), and verticalization of the DITS (right-hand arrow). The right hippocampus does not exhibit clear-cut features of HIMAL. (**B**) An example of a subject with normal hippocampi. The right-hand parts of **A** and **B** provide demonstrations of the three quantitative measures implemented in the study: diameter ratio, i.e. *z* divided by *x*; DITS height ratio, i.e. *z* divided by *a*, or value equal to zero in the absence of overlap between DITS and hippocampus, resulting in no measurable *a*; and parahippocampal angle, for a hippocampus with HIMAL (**A**) and for a normal hippocampus (**B**). See main text for details.

Anonymized T_1_-weighted images were visually inspected by two trained raters (L.C., F.X.) based on a global impression, accounting for shape and orientation of the hippocampus, depth and verticalization of the collateral and/or occipito-temporal sulcus, as well as appearance of the subiculum and parahippocampal gyrus (Bernasconi *et al.*, 2005; Cury *et al.*, 2015; Tsai *et al.*, 2016). Each hippocampus was rated as normal, borderline or with HIMAL by each investigator independently; final diagnosis was reached via consensus. As a further means to formally assess inter-rater reliability and validate qualitative assessments, formal coding of the three criteria found to be highly associated with HIMAL by Tsai *et al.* (2016) was determined as follows: (i) hippocampal shape: normal, borderline or rounded/pyramidal; (ii) verticalization of the dominant inferior temporal sulcus (DITS), the most prominent between the collateral sulcus and the occipito-temporal sulcus: definite, borderline or normal; and (iii) shape of the lateral hippocampal aspect: curved, borderline or flattened (Fig. 1).

Using ITK-SNAP (3.6.0), we also obtained quantitative parameters (Fig. 1), and chose the three features previously found to be significantly associated with HIMAL by Tsai *et al.* (2016): (i) hippocampal diameter ratio, representing the hippocampal height divided by its width, measured on the slice where the hippocampal shape appears most affected; (ii) DITS height ratio, consisting of a ratio between the distance from the inferior hippocampal margin to the superior limit of the DITS, and the hippocampal vertical diameter, estimated on the slice where the sulcus is deepest (in case of no overlap between DITS and hippocampus, this measure is equal to zero); and (iii) parahippocampal angle, measured on the first coronal slice including the hippocampal body, and consisting of the angle between the ascending and descending white matter branches of the parahippocampal gyrus.

### Neuropsychological data

The National Adult Reading Test (NART) (Nelson and Willison, 1991) provided a measure of estimated intellectual level (IQ). The List Learning and Design Learning subtests of the Adult Memory and Information Processing Battery (AMIPB) addressed learning and recall of verbal and visuo-spatial cues, respectively. During the verbal learning task, the participants were read a list of 15 words to be subsequently recalled, which was repeated for five consecutive trials (A1–A5). After a short period of distraction, they were asked to recall again the words of the list (A6). A similar test, implementing an abstract design that subjects were required to draw, was utilised to obtain measures of visuo-spatial learning (A1–A5; A6). These measures were previously shown to be sensitive to the integrity of mesiotemporal structures (Baxendale *et al.*, 2006).

### Statistical analysis of hippocampal measures and neuropsychometry

Hippocampal measures and behavioural data were analysed using SPSS Statistics 24.0 (IBM). Inter-rater reliability for (i) pre-consensus diagnosis of HIMAL; (ii) qualitative; and (iii) quantitative assessments of hippocampal features were conducted using weighted Cohen’s kappa or ICC, as appropriate. Statistics for items (ii) and (iii) were conducted on a randomly selected data subset (44 hippocampi, 30% of the sample). Chi-squared test was used for categorical data. Multivariate/univariate ANOVA or Kruskal-Wallis test were used for parametric or non-parametric data, with *post hoc* Bonferroni corrections for multiple comparisons unless otherwise stated. Binomial logistic regression with Firth’s correction was implemented to identify categorical factors associated with HIMAL, while the relationship between HIMAL, hippocampal volume, group allocation, gender, handedness and neuropsychological test scores was assessed with multiple regression analyses.

### Functional imaging

#### Task specifics

During a single scanning session, a total of 70 visual and 70 verbal items, grouped in seven blocks comprising 10 visual (faces) and 10 verbal (words) items each, were visually presented via an MRI compatible screen. Each item was displayed for 3 s within 30-s blocks. A 15-s cross-hair fixation block (control condition) was intercalated every two task blocks. We used a different inter-stimulus interval (3 s) compared to our repetition time of 2.5 s to introduce jitter and ensure random sampling. The visual blocks included a combination of neutral and fearful non-famous faces, unfamiliar to the subjects, while single concrete nouns (including emotionally adverse words such as ‘cancer’ or ‘famine’) were used during the verbal blocks. The participants were explicitly instructed to memorize the items for subsequent out-of-scanner recall. A deep encoding task (Craik, 2002), involving a subjective decision on whether each stimulus was pleasant or unpleasant, was performed via a joystick. During the recall task, the items presented previously were intermixed with additional 50% novel stimuli in random order, and the subjects used a button box to indicate whether items were remembered or novel. Recognition accuracy was calculated as true positives minus false positives for words and faces separately. Functional MRI data were available for analysis in 28 patients, 12 siblings and 18 controls.

#### Data processing and analysis

Functional MRI data were analysed using SPM8 version 5236 software (http://www.fil.ion.ucl.ac.uk/spm/). Images were realigned for motion correction, spatially normalized to a scanner- and acquisition-specific echo-planar imaging template in Montreal Neurological Institute (MNI) space, resampled to isotropic 3 × 3 × 3 mm voxels, and smoothed with a Gaussian kernel of 8 mm full-width at half-maximum. An event-related analysis was used to specifically investigate subsequent memory effects, by comparing encoding-related responses for subsequently remembered stimuli against those for forgotten stimuli using a two-level random-effect model. Trial-specific delta functions were convolved with the haemodynamic response function and its temporal and dispersion derivatives. For each participant, each of the four event types, namely words remembered (WR) or forgotten (WF) and faces remembered (FR) or forgotten (FF), was modelled by a regressor of interest, and motion parameters were included as regressors of no interest. Contrasts were created for each subject to address verbal or visual subsequent memory, defined by WR-WF and FR-FF, respectively. Having recognized the full item list, three patients with JME and two control subjects were excluded from the verbal functional MRI analysis. At the second level, one-sample *t*-tests addressed the main effects of each contrast across all subjects, and separately within each group (JME, SIB or healthy controls). Group comparisons were conducted with one-way ANOVA. Further subgroup analyses were conducted for the JME group only, because of sample size constraints, to compare JME with or without HIMAL and controls. In accordance with previous literature, peak-level mesiotemporal activations were considered significant at a threshold of *P < *0.05, corrected for multiple comparisons (FWE) using a small-volume correction with a 12-mm diameter sphere, centred at the location of the activation maximum in the mesiotemporal regions of interest, hereafter reported as FWE-svc (Sidhu *et al.*, 2013; Zeidman *et al.*, 2015). Extra-mesiotemporal activations were considered statistically significant at a threshold of *P < *0.05, FWE-corrected voxel-wise across the whole brain. Exploratory assessments were conducted up to a threshold of *P < *0.01, uncorrected, using a cluster extent threshold of 10 voxels for display purposes.

### Structure-function relations

We investigated correlations between memory-related activations and morphological markers of hippocampal positioning. The measures of hippocampal diameter ratio, DITS height ratio and parahippocampal angle were entered in a principal component analysis. For left hippocampal parameters, the first principal component (eigenvalue: 2.63, accounting for 87.5% of the total variance) was considered as a composite marker of left hippocampal positioning. Similarly, a component with eigenvalue of 2.12, explaining 70.7% of the variance, was regarded as an overall measure of right hippocampal positioning. More positive values assumed by the latter variables related to more malrotated hippocampi, i.e. presenting with larger values for hippocampal diameter ratio and DITS height ratio, and a more acute parahippocampal angle. Across all subjects, multiple regression models were implemented within SPM to assess correlations between the composite marker of left or right hippocampal positioning and mesiotemporal activation during successful verbal or visual encoding, respectively. Analyses were also repeated for a subgroup composed of patients with JME and their siblings. Ipsilateral hippocampal volume was used as nuisance regressor in all models, to detect associations specific to hippocampal positioning. Mesiotemporal correlational activations were considered significant at a threshold of *P < *0.05, corrected for multiple comparisons (FWE) using a small-volume correction with a 12-mm diameter sphere, centred at the location of the mesiotemporal activation maximum (Bonelli *et al.*, 2012; Sidhu *et al.*, 2013). Extra-mesiotemporal correlational activations are reported for completeness, and considered statistically significant at a threshold of *P < *0.05, FWE-corrected voxel-wise across the whole brain.

### Data availability

The data supporting the findings of this study are available from the corresponding author upon reasonable request. They are not publicly available because of ethical restrictions.

## Results

### Hippocampal volumetry

There was a significant group effect regarding left hippocampal volume [JME/siblings/healthy controls, mean (SD): 2743 mm^3^ (247)/ 2663 mm^3^ (207)/ 2907 mm^3^ (220); one-way ANOVA: *F*(2,70) = 5.47, *P = *0.006, partial η^2 ^= 0.14]. *Post hoc* tests (Bonferroni-corrected) indicated that both JME patients (*P = *0.038, Cohen’s *d* = 0.7) and their siblings (*P = *0.007, Cohen’s *d* = 1.14) had a smaller left hippocampus than controls. *Post hoc* comparison of JME and siblings was not statistically significant (*P = *0.76). Repeat models using age, gender and handedness as covariates, along with further sensitivity analyses accounting for outliers, showed convergence of volumetric measures for JME and siblings, while confirming differences against controls (Supplementary material). There were no significant between-group differences for right hippocampal volume [JME/siblings/healthy controls, mean (SD): 2859 mm^3^ (285)/2870 mm^3^ (161)/2950 mm^3^ (211); one-way ANOVA: *F*(2,70) = 0.95, *P = *0.39, partial η^2 ^= 0.03]. In patients with JME, neither left nor right hippocampal volume correlated with disease duration (left/right hippocampus, Pearson’s *r*: 0.03/−0.24, *P = *0.86/0.16) or age at disease onset (*r = *0.06/0.03, *P = *0.73/0.87).

### Hippocampal malrotation

#### Qualitative analysis

Based on the criteria outlined previously, a consensus diagnosis of unilateral or bilateral HIMAL was made in a total of 30 subjects (left/right/bilateral malrotation: 22/3/5; examples are shown in Supplementary Fig. 1). In line with previous literature (Cury *et al.*, 2015; Tsai *et al.*, 2016), malrotation was significantly more frequent for the left than for the right hippocampus (Pearson χ^2 ^= 13.57, *P < *0.001, Cramer’s V = 0.31). Hippocampal shape, lateral aspect and verticalization of DITS were all significantly associated with HIMAL (*P < *0.0001; Supplementary Table 1). Nineteen patients with JME (51.4%), eight JME siblings (50%) and three healthy controls (15%) presented with unilateral or bilateral HIMAL (left/right/bilateral malrotation: 13/2/4 for JME patients, 6/1/1 for siblings, 3/0/0 in controls). There was a significant association between study group and malrotation of at least one hippocampus (Pearson χ^2 ^= 7.76, *P = *0.021). *Post hoc* tests indicated a higher frequency of HIMAL in JME and siblings compared with controls (JME versus controls: χ^2 ^= 7.24, *P = *0.014, Cramer’s V = 0.36; siblings versus controls: χ^2 ^= 5.13, *P = *0.046, Cramer’s V = 0.38; Bonferroni-corrected based on two *post hoc* tests).

A logistic regression model, probing the effects of group, gender and handedness on the likelihood of HIMAL, was statistically significant [χ^2^(4) = 17.8, *P = *0.001]. Group was a significant predictor [W(2) = 9.58, *P = *0.008], with patients with JME being 14.6 times [W(1) = 9.45, *P = *0.002] and JME siblings being 10.6 times [W(1) = 6.44, *P = *0.011] more likely than controls to exhibit HIMAL. Gender was also a significant predictor [W(1) = 5.86, *P = *0.015], with malrotation being 4.4 times more likely in males than females, while handedness was not significant [W(1) = 0.33, *P = *0.57]. Inter-rater reliability of qualitative features was high, with κ = 0.77/0.80/0.80 for shape, verticalization of DITS and lateral hippocampal margin, and κ = 0.90 for between-investigator agreement regarding HIMAL diagnosis before consensus discussion.

#### Quantitative analysis

Comparison of hippocampi with and without malrotation, irrespective of group allocation, revealed significant differences in hippocampal diameter ratio, DITS height ratio and parahippocampal angle (all *P < *0.0001; Supplementary Table 1), corroborating high discrimination with the chosen criteria. MANOVA, testing for group differences across hippocampal quantitative measures, was statistically significant [Pillai’s trace = 0.36, *F*(12,132) = 2.44, *P = *0.007, partial η^2 ^= 0.18]. Univariate analyses of variance for the items of the MANOVA are reported in Table 2. We found significant group effects for: (i) left hippocampal diameter ratio, with *post hoc* tests showing higher values in JME and siblings compared to controls, indicative of a more verticalized hippocampus (corrected, Tukey’s range test); and (ii) right hippocampal diameter ratio, with *post hoc* tests showing effects in the same direction as for the left hippocampus. For both comparisons, there were no differences between JME and siblings. We also found significant group effects for (iii) left parahippocampal angle, with *post hoc* tests indicating a more acute angle in JME patients compared with controls only. There were no significant intergroup differences for left and right hippocampal DITS height ratio and right parahippocampal angle. ICCs for hippocampal diameter ratio, DITS height ratio and parahippocampal angle were 0.88, 0.91 and 0.83, respectively, indicating excellent inter-rater agreement.

**Table 2:** MANOVA on quantitative hippocampal measures

Test	Test statistic	*P*-value	Mean (SD)	*Post hoc P*-value
Diameter ratio, left hippocampus (%)	*F*(2,70) = 3.89	**0.025**	JME: 75.3 (15.9)	JME/CTR: **0.044**
			SIB: 77.5 (18.5)	SIB/CTR: **0.044**
			CTR: 65.0 (10.4)	JME/SIB: 0.89
Diameter ratio, right hippocampus (%)	*F*(2,70) = 4.27	**0.018**	JME: 69.6 (13.0)	JME/CTR: **0.025**
			SIB: 70.4 (8.7)	SIB/CTR: **0.047**
			CTR: 61.9 (4.7)	JME/SIB: 0.97
DITS height ratio, left hippocampus (%)	*F*(2,70) = 2.36	0.10	JME: 47.8 (20.8)	–
			SIB: 39.6 (25.7)	
			CTR: 36.0 (15.4)	
DITS height ratio, right hippocampus (%)	*F*(2,70) = 2.90	0.062	JME: 28.0 (18.1)	–
			SIB: 24.5 (20.4)	
			CTR: 16.5 (12.5)	
Parahippocampal angle, left hippocampus (degrees)	*F*(2,70) = 3.37	**0.04**	JME: 109.5 (16.3)	JME/CTR: **0.031**
			SIB: 112.6 (15.3)	SIB/CTR: 0.30
			CTR: 120.0 (10.3)	JME/SIB: 0.75
Parahippocampal angle, right hippocampus (degrees)	*F*(2,70) = 1.11	0.34	JME: 117.3 (13.2)	–
			SIB: 120.6 (12.4)	
			CTR: 121.9 (8.2)	

Univariate analyses for items of the MANOVA on hippocampal quantitative measures. *Post hoc* evaluations (corrected, Tukey's range test; fifth column) were carried out on statistically significant items of the MANOVA. Values in bold indicate statistically significant *P*-values.

CTR = healthy controls; SIB = unaffected JME siblings.

Further analyses assessing accuracy of hippocampal morphometric measures in discriminating study subgroups are reported in Supplementary material.

#### Relationship with hippocampal volumetry and clinical characteristics

Considering patients with JME and their siblings, a regression model with corrected hippocampal volume as dependent variable and side of the hippocampus (left or right), group allocation, gender, handedness and HIMAL as independent variables was significant [*F*(5,98) = 2.64, *P = *0.028, *R*^2^* = *0.12], but identified a significant effect of hippocampal side only (beta = 0.23, *t* = 2.25, *P = *0.027), with right hippocampus being associated with a larger volume than the left. Though HIMAL was related to slightly smaller hippocampal volumes, the latter difference was not statistically significant (beta = −0.12, t = −1.1, *P = *0.27). In patients with JME, HIMAL was not associated with a history of febrile seizures (Fisher’s Exact test, *P = *1.0), with an earlier seizure onset (standardized Mann-Whitney U-test = −0.603, *P = *0.57), or worse seizure control (i.e. seizures in the last year; χ^2 ^= 0.32, *P = *0.86).

### Neurobehavioural data

There were no significant group differences for general intellectual level (NART IQ), measures of verbal learning (A1–A5), verbal recall (A6) and visuo-spatial learning (A1–A5). Kruskal-Wallis test highlighted differences for visuo-spatial recall (A6), although corrected *post hoc* tests were not statistically significant (Table 1). A regression analysis to ascertain the influence of left HIMAL, group allocation, and age on memory scores (verbal learning/recall) was overall not significant, and did not substantiate an effect of HIMAL (beta = 0.10/0.12, t = 0.79/0.84, *P = *0.44/0.41 for verbal learning/recall, respectively). Analyses addressing factors influencing visuo-spatial memory performance could not be carried out owing to the scarcity of right HIMAL cases. There were no significant differences for verbal and visuo-spatial learning scores between seizure free and non-seizure free JME patients (all *P* > 0.05, Mann-Whitney U-test). Recognition accuracy of the memory encoding functional MRI task did not differ among groups (Table 1).

### Mesiotemporal function

#### Verbal memory functional MRI

**Figure 2:**
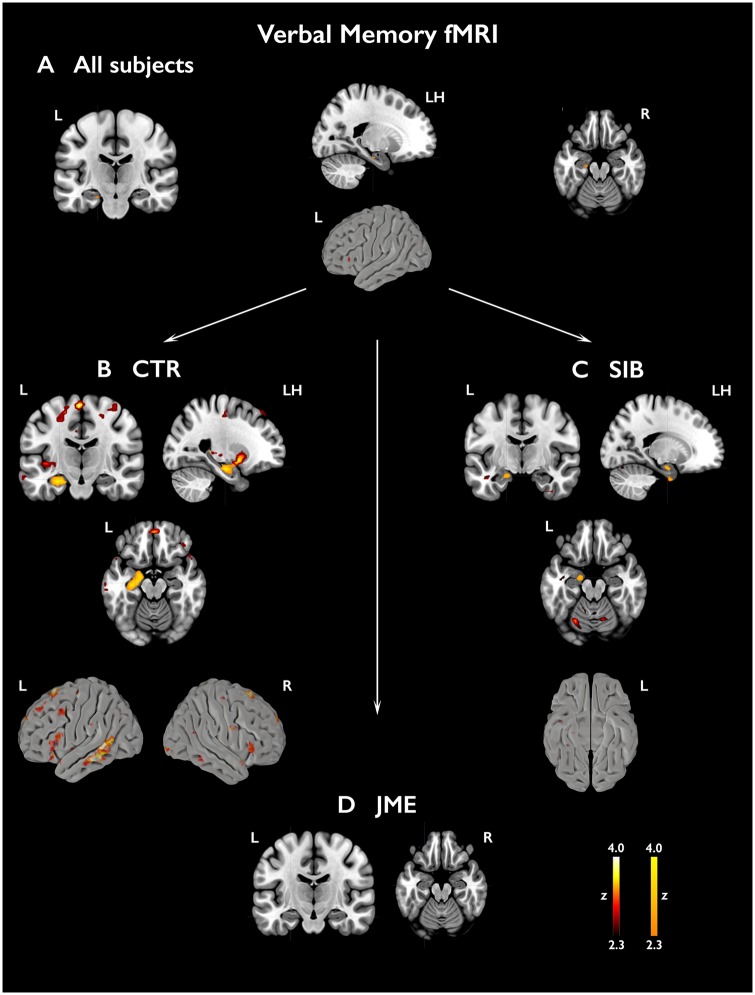
**Activations related to subsequent verbal memory.** Activations in relation to subsequently remembered verbal items (WR-WF) are shown across all subjects (**A**) and separately for each group [**B**, controls; **C**, siblings (SIB); **D**, patients with JME]. Statistical maps for mesiotemporal activations (orange-yellow scale) show voxels included in the 12-mm diameter spherical regions of interest used for multiple comparison correction, centred on local maxima, where peak-level significance at *P < *0.05-FWE corresponds to z-scores >2.3). Extra-mesiotemporal activations are displayed at an uncorrected threshold (*P < *0.01, cluster extent threshold of 10 voxels; ‘hot’ colour scale). ‘LH’ refers to a sagittal section of the left hemisphere. Colour bars reflect z-score scales for mesiotemporal (*right*) and extra-mesiotemporal activations (*left*). MNI coordinates and parameter estimates are provided in Supplementary Table 2.

**Figure 3:**
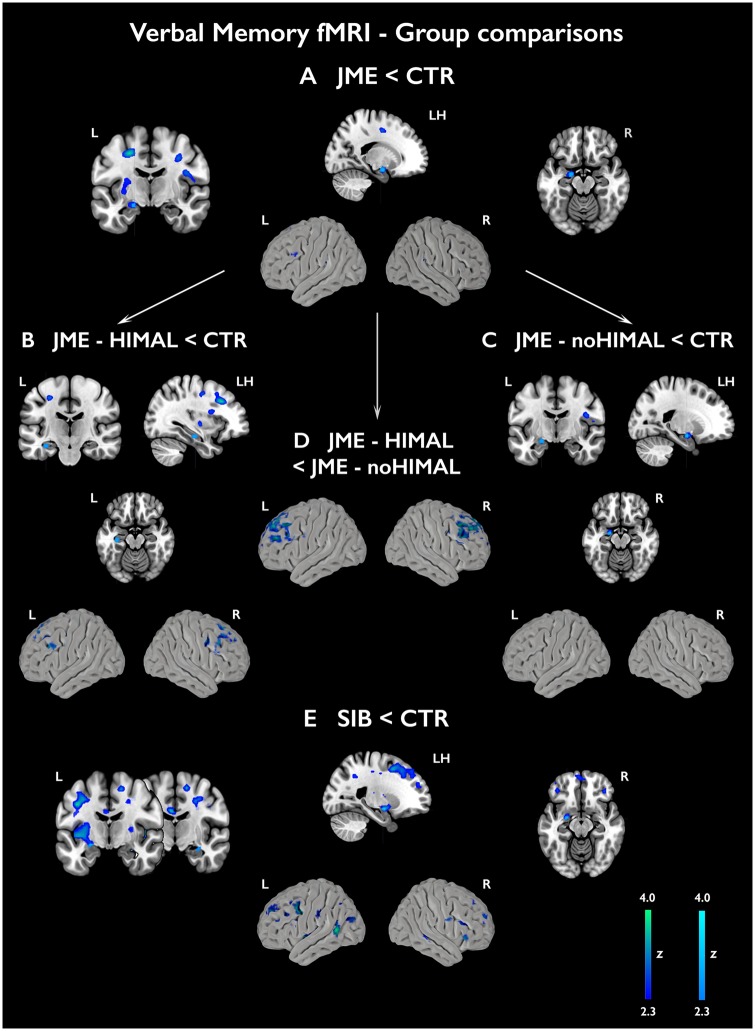
**Group comparisons for subsequent verbal memory activations.** The figure shows group comparisons for activation related to subsequently remembered verbal items (WR-WF) in patients with JME compared with controls (**A**), along with subgroup analyses for JME with HIMAL (**B**) and JME without HIMAL versus controls (**C**), and for the direct comparison of JME with and without HIMAL (**D**). Comparison of JME siblings against controls is shown in **E**. Statistical maps for mesiotemporal activations (cyan scale) show voxels included in the 12-mm diameter spherical regions of interest used for multiple comparison correction, where peak-level significance at *P < *0.05-FWE corresponds to z-scores >2.3). Extra-mesiotemporal activations are displayed at an uncorrected threshold (*P < *0.01, cluster extent threshold of 10 voxels; blue-green colour scale). ‘LH’ refers to a sagittal section of the left hemisphere. Colour bars reflect z-score scales for mesiotemporal (*right*) and extra-mesiotemporal activations (*left*). MNI coordinates are provided in Supplementary Table 3.

Across all subjects, the verbal memory task elicited the activation of the left hippocampus (*P < *0.05, FWE-svc). In healthy controls, robust effects were identified within left hippocampus, amygdala and parahippocampal gyrus (*P < *0.05, FWE-svc). Left hippocampal and parahippocampal activation was observed in siblings (*P < *0.05, FWE-svc), while hippocampal activation failed to reach statistical significance in the JME group (Fig. 2 and Supplementary Table 2). In controls, uncorrected left-lateralised effects (all *P < *0.001) were detected in extra-mesiotemporal regions, including middle temporal, inferior, middle and superior frontal gyrus, orbitofrontal cortex and putamen (Fig. 2 and Supplementary Table 2). As for mesiotemporal activation, extra-temporal effects were scarce in JME and siblings. An *F*-test in a one-way ANOVA (Supplementary Table 3) indicated significant between-group differences in mesiotemporal activation, and uncorrected group differences for bilateral fronto-temporal cortices. Pair-wise comparisons against controls (Fig. 3 and Supplementary Table 3) revealed significantly lower left amygdalo-hippocampal activation in JME patients (*P = *0.019, FWE-svc) and reduced left amygdalo-hippocampal and right hippocampal activation in JME siblings (left local maximum: *P = *0.009, FWE-svc; right local maximum: *P = *0.049, FWE-svc). Analyses controlling for left hippocampal volume did not affect significance of left mesiotemporal comparisons (Supplementary Table 3). At uncorrected thresholds, there was reduced activation of fronto-temporal cortices in JME and siblings (Fig. 3 and Supplementary Table 3).

Subgroup analyses showed that lower activation in JME-HIMAL compared with controls was confined to the left hippocampal body (*P = *0.025, FWE-svc), whereas patients with JME and normal hippocampi exhibited lower amygdala and anterior hippocampal activation compared to controls (*P = *0.035, FWE-svc). Repeat models with left hippocampal volume as covariate produced virtually identical results (Supplementary Table 3). We also detected lower recruitment of the middle frontal gyrus in JME-HIMAL compared to JME with normal hippocampi (right/left: *P < *0.031, whole-brain FWE-corrected/*P < *0.001 uncorrected, respectively). A similar pattern was evidenced for JME-HIMAL against controls at *P < *0.001, uncorrected (Fig. 3).

#### Visual memory functional MRI

**Figure 4:**
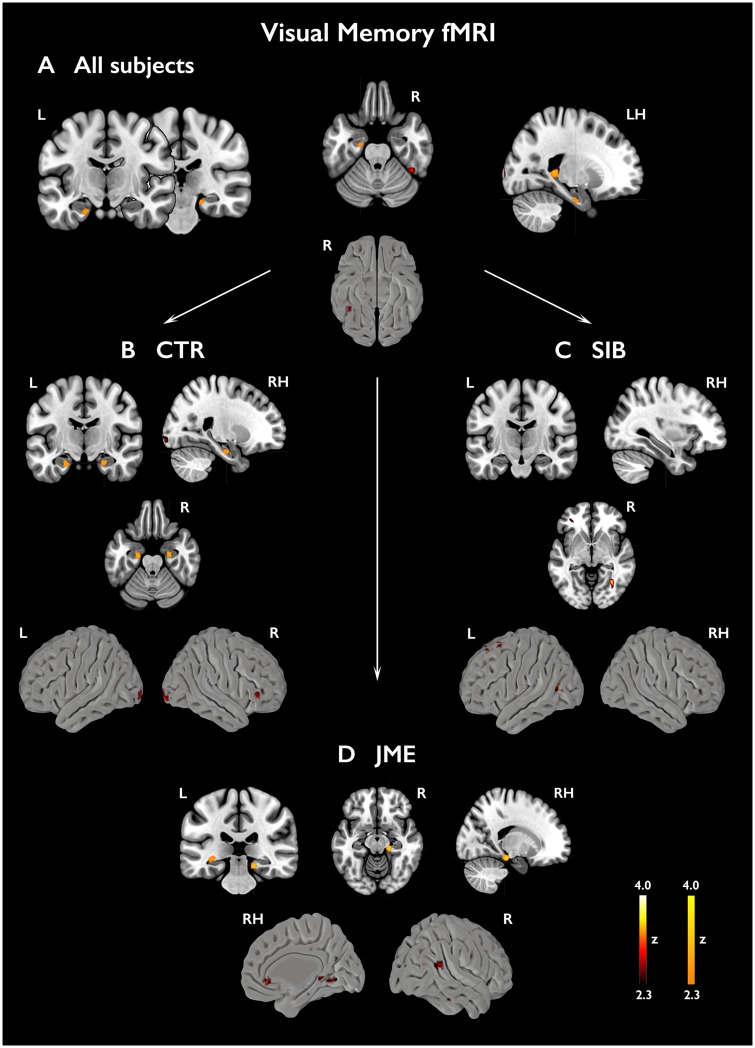
**Activations related to subsequent visual memory.** Mesiotemporal activations in relation to subsequently remembered visual items (FR – FF) are shown across all subjects (**A**) as well as separately for each group [**B**, controls; **C**, siblings (SIB); **D**, patients with JME]. Statistical maps for mesiotemporal activations (orange-yellow scale) show voxels included in the 12-mm diameter spherical regions of interest used for multiple comparison correction, where peak-level significance at *P < *0.05-FWE corresponds to z-scores >2.3). Extra-mesiotemporal activations are displayed at an uncorrected threshold (*P < *0.01, cluster extent threshold of 10 voxels; *‘hot’* colour scale). Colour bars reflect z-score scales for mesiotemporal (*right*) and extra-mesiotemporal (*left*) activations. ‘LH/RH’ refer to sagittal sections of the left/right hemisphere, respectively. MNI coordinates and parameter estimates are provided in Supplementary Table 4.

**Figure 5:**
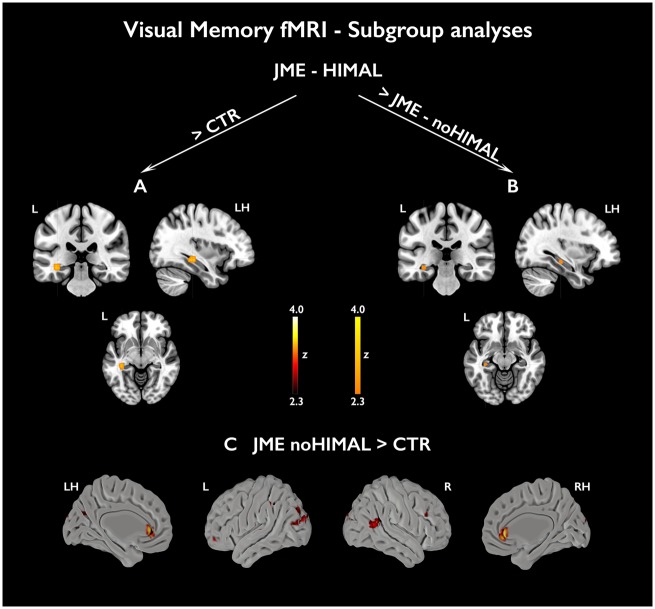
**Subgroup analyses for subsequent visual memory.** The figure shows subgroup analyses comparing mesiotemporal activation for subsequently remembered visual items (FR − FF) in JME with and without HIMAL against healthy controls (**A** and **C**, respectively), along with the direct contrast of JME with and without HIMAL (**B**). Statistical maps for mesiotemporal activations (orange-yellow scale) show voxels included in the 12-mm diameter spheres used for multiple comparison correction, where peak-level significance at *P < *0.05-FWE corresponds to z-scores >2.3. Extra-mesiotemporal activations are displayed at an uncorrected threshold (*P < *0.01, cluster extent threshold of 10 voxels; ‘hot’ colour scale). ‘LH/RH’ refers to a sagittal section of the left/right hemisphere, respectively. Colour bars reflect z-score scales for mesiotemporal (*right*) and extra-mesiotemporal (*left*) activations. MNI coordinates are provided in Supplementary Table 5.

Across all subjects, significant activations related to successful visual encoding were found in left anterior and posterior hippocampus and right parahippocampal gyrus (Fig. 4 and Supplementary Table 4). Robust effects were identified within bilateral anterior hippocampi in healthy controls (*P < *0.05, FWE-svc). Activation of right parahippocampal gyrus and left posterior hippocampus (*P < *0.05, FWE-svc) was identified in patients with JME, but there was no suprathreshold right hippocampal activation. Siblings exhibited right anterior and left posterior hippocampal activations, which were not statistically significant. Across all groups, extra-mesiotemporal activation was scarce (Fig. 4 and Supplementary Table 4) An *F*-test in a one-way ANOVA did not substantiate significant between-group differences. Subgroup analyses comparing JME with or without HIMAL and controls (Fig. 5 and Supplementary Table 5) revealed higher left posterior hippocampal activation in JME with HIMAL than controls (*P = *0.007, FWE-svc) and JME with normal hippocampus *P = *0.034, FWE-svc). Covarying for left hippocampal volume did not affect the results (Supplementary Table 5). There were no significant mesiotemporal differences between JME without HIMAL and controls, while extra-temporal uncorrected differences included higher anterior cingulate activation in JME without HIMAL.

### Structure-function relations

**Figure 6:**
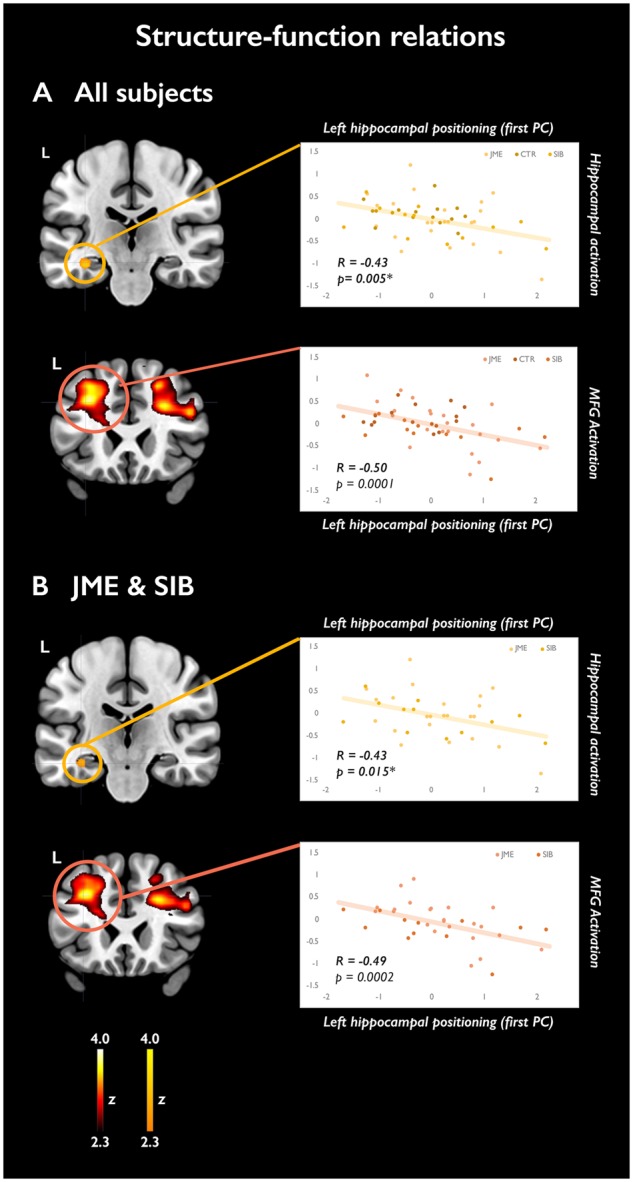
**Structure-function relations.** The scatterplots highlight the association between the composite morphological indicator of left hippocampal positioning and activation of the left hippocampus (orange-yellow scale) and middle frontal gyrus (‘hot’ colour scale) for successfully encoded verbal items. (**A**) Analysis of all study subjects. (**B**) Analysis of a subgroup composed of patients with JME and their siblings. Local activation maxima, corresponding to the area of maximal correlation between structural and functional metrics, are shown on coronal sections. Maps for mesiotemporal effects show voxels included in the 12-mm diameter sphere centred on the local maximum, where peak-level significance at *P < *0.05-FWE corresponds to a z-score >2.3. Extra-mesiotemporal activations are displayed at an uncorrected threshold (*P < *0.01, cluster extent threshold of 10 voxel), corresponding to z scores >2.3. Colour-bars reflect z-score scales for mesiotemporal and extra-mesiotemporal activations. The *P*-values reported on the scatterplots for hippocampal effects are small-volume corrected for multiple comparisons (asterisk); the *P*-values for middle frontal effects are uncorrected for multiple comparisons. MNI coordinates are provided in Supplementary Table 6.

For successful verbal encoding, left hippocampal activation was negatively associated with the composite index of left hippocampal positioning (*P = *0.005, FWE-svc; Fig. 6 and Supplementary Table 6). Findings remained unchanged when additionally controlling for group allocation. Repeat models, assessing structure-function correlations in a subgroup composed of JME patients and siblings, produced similar results (*P = *0.015, FWE-svc; Fig. 6 and Supplementary Table 6). Uncorrected negative associations were also detected between hippocampal positioning and bilateral middle frontal activation, with peaks on the left, both for the whole sample and in the JME-sibling subgroup (all *P < *0.001, uncorrected; Fig. 6 and Supplementary Table 6).

No significant correlations were detected between mesiotemporal activations for visual encoding and the composite indicator of right hippocampal positioning.

## Discussion

We detected abnormal morphometric features of the hippocampus, including reduced left hippocampal volume, higher prevalence of malrotation, and more marked vertical orientation. Assessment of mesiotemporal function using neuropsychological tests and functional MRI revealed no overt impairment of verbal and non-verbal memory performance, but highlighted atypical patterns of mesiotemporal activation in JME, particularly prominent in the subgroup with HIMAL. Most of the above traits were substantiated in unaffected JME siblings, suggesting a heritable basis, and pointing to the hippocampus as a structure affected by genetic variants predisposing to JME.

### Hippocampal volume loss in JME and unaffected siblings

Previous analyses addressing brain morphology in JME identified mesial or lateral frontal abnormalities (Woermann *et al.*, 1999; O’Muircheartaigh *et al.*, 2011; Alhusaini *et al.*, 2013), suggesting either reduced or increased cortical volume or thickness, along with atrophy of thalami and basal ganglia and impaired fronto-cortico-subcortical connectivity (Keller *et al.*, 2011; O’Muircheartaigh *et al.*, 2012; Perani *et al.*, 2018). Several reports detected morphological abnormalities encompassing lateral temporal neocortices (Ronan *et al.*, 2012; Lin *et al.*, 2014), while data regarding the involvement of mesiotemporal structures in JME are discordant (Lin *et al.*, 2013; Kim *et al.*, 2015, 2018). Our findings indicate left hippocampal volume loss (5–8%) in both patients with JME and their siblings compared with controls. Siblings exhibited slightly smaller mean volumes than patients, but intergroup differences were not statistically significant. Sensitivity analyses showed convergence of left hippocampal volumetric values for patients and siblings, and further divergence of both from controls. Thus, our findings do not substantiate volumetric differences between patients and their siblings. In the JME group, hippocampal volume was not influenced by epilepsy duration or age at disease onset.

Evidence of hippocampal volume reduction, more marked on the left, was also reported in genetic generalized epilepsy with generalized tonic-clonic seizures only (Zhou *et al.*, 2015). The aetiology of hippocampal changes in genetic generalized epilepsy syndromes, such as JME, remains debated. Focal epileptiform waveforms, including temporal spikes, are described in ∼35% of JME cases (Aliberti *et al.*, 1994), and EEG source analyses identified basal or mesial temporal sources (Holmes *et al.*, 2010). Nonetheless, EEG-functional MRI analyses in mixed genetic generalized epilepsy groups and in JME do not suggest the involvement of the hippocampus (Hamandi *et al.*, 2006; Dong *et al.*, 2016). With our data, we cannot establish whether hippocampal abnormalities in JME point to ictogenic networks involving the mesiotemporal structures. The absence of correlations with disease-related variables, along with the finding of atrophy in unaffected JME siblings, however, concurs with the view that hippocampal volume loss may represent a genetically determined trait. Previous literature indicates high heritability of global hippocampal volumes and individual hippocampal subfields (Blokland *et al.*, 2012; Whelan *et al.*, 2015). Higher vulnerability of the left hippocampus, as found here, may relate to asymmetric gene expression profiles underlying hippocampal development (Sun *et al.*, 2005; Moskal *et al.*, 2006). Moreover, subtle hippocampal abnormalities, in the same range (4–8%) as those shown here for JME, were reported for disorders with childhood- or adolescence-onset and abnormal neurodevelopment, including attention deficit and hyperactivity disorder (Hoogman *et al.*, 2017) and schizophrenia, where they additionally involved first-degree relatives (Moran *et al.*, 2013). Similar to our study, one investigation in schizophrenia reported smaller left hippocampal volumes in siblings relative to patients (Tepest *et al.*, 2003). Such findings might stem from higher homogeneity in the sibling group, sampling error, or a combination of these.

By detecting common left hippocampal volume loss in patients with JME and their siblings, our findings may thus point to genetic vulnerability, identifying a novel anatomical abnormality associated with the genetic risk for JME. Overall, it is tempting to consider subtle hippocampal abnormalities in JME and siblings as reflective of genetically-driven altered neurodevelopment mechanisms. The latter appear common features of neuropsychiatric diseases with complex multi-factorial aetiologies.

### More than volume: HIMAL as a novel JME endophenotype?

HIMAL, also termed incomplete hippocampal inversion, refers to an atypically shaped hippocampus, with round or pyramidal configuration, loss of the lateral convexity and verticalization of the collateral sulcus, owing to incomplete mesiotemporal infolding during prenatal neurodevelopment (Bernasconi *et al.*, 2005; Bajic *et al.*, 2008). HIMAL appears common in patients with malformations of cortical development (MCD) causing epilepsy (Baulac *et al.*, 1998; Bernasconi *et al.*, 2005). The prevalence of HIMAL in MCD is higher than in temporal lobe epilepsy, where its lateralization may not overlap with that of the epileptogenic focus, suggesting independent aetiologies (Tsai *et al.*, 2016). In single-centre studies, prevalence in healthy controls was 10–24% (Bernasconi *et al.*, 2005; Bajic *et al.*, 2008; Tsai *et al.*, 2016), while a multicentre investigation on ∼2000 healthy subjects detected HIMAL in 17% and 6% of left and right hippocampi, respectively (Cury *et al.*, 2015).

It is debated whether HIMAL may represent a pathological entity. Prevalence in unaffected control populations is not negligible, and relates to abnormalities of temporal sulcal morphometry (Cury *et al.*, 2015), whilst the frequent co-existence with overtly anomalous brain development and/or epilepsy is equally unquestionable. Recent evidence points to more frequent findings of HIMAL in cases of febrile status epilepticus compared to simple febrile seizures (Chan *et al.*, 2015), and hippocampal maldevelopment was documented for sudden unexplained death in childhood (Kinney *et al.*, 2009; Hefti *et al.*, 2016). Overall, these multiple lines of evidence suggest that HIMAL may not be an entirely benign finding.

Here, we detected a significantly higher occurrence of HIMAL both in JME and siblings, affecting about 50% of individuals in each subgroup, as opposed to a prevalence of 15% healthy controls, which mirrors previous observations. Logistic regression modelling, controlling for gender and handedness, corroborated significantly higher odds of HIMAL in JME and siblings. Moreover, comparisons regarding the horizontal to vertical hippocampal diameter ratio, expressing a quantitative measure of vertical orientation, identified significantly higher values in the whole JME and sibling groups for both left and right hippocampi, capturing a group-level phenomenon not easily addressed by qualitative criteria. The left parahippocampal angle, representing another marker of vertical orientation, was more acute in patients with JME and their siblings, though *post hoc* tests were significant for the JME group only. As previously reported (Cury *et al.*, 2015; Tsai *et al.*, 2016), HIMAL was more frequent in left than right hippocampi. This relates to evidence of faster right-sided hippocampal development (Bajic *et al.*, 2012), and may occur as a consequence of asymmetric gene expression (Sun *et al.*, 2005; Moskal *et al.*, 2006).

Collectively, this is the first demonstration of morphometric anomalies affecting the hippocampal formation in JME. Similar prevalence of HIMAL and overlapping hippocampal morphometric patterns in unaffected JME siblings indicate trait heritability, suggesting a genetic basis. Along with hippocampal volume loss, hippocampal malpositioning can thus be construed as a novel imaging intermediate phenotype (endophenotype) of JME. As HIMAL implicates incomplete mesiotemporal infolding during gestation, we interpret abnormalities of hippocampal morphometry as the reflection of mesiotemporal vulnerability during prenatal neurodevelopment. Our findings provide novel insights into the structural neural correlates of the genetic risk for JME, suggesting the involvement of regions beyond the classically implicated fronto-cortical networks.

In accordance with the definition of endophenotype (Gottesman and Gould, 2003), the reported mesiotemporal abnormalities may be associated with the JME phenotype at the population level, but not sufficient to develop the disease. The lack of a relation between disease variables and HIMAL among JME patients suggests that hippocampal anomalies may not represent suitable markers of epilepsy-related symptom severity in disease cases, indicating likely distinct genetic underpinnings. However, the quantitative traits with endophenotypic potential identified in our study, hippocampal volume and diameter ratio, showed high discrimination of JME patients and of a combined JME-sibling subgroup from controls, both individually and via a composite construct. These findings support suitability of the latter as quantitative traits to assist future large-scale investigations into the genetic substrates of JME, paralleling initiatives in psychiatric research (Potkin *et al.*, 2009; Blokland *et al.*, 2018).

Pointing to a prenatal neurodevelopmental basis, our findings dovetail with current theories on the aetiology of JME, conceptualized as a polygenetic condition with high heterogeneity (Wolf *et al.*, 2015). Whether abnormalities predisposing to JME may overlap with determinants of hippocampal development under a unifying genetic framework remains undetermined. Several genetic loci have been associated with hippocampal volume (Stein *et al.*, 2012; Hibar *et al.*, 2017), but the genetic underpinnings of HIMAL are unclarified. Frequent incidence of HIMAL (64%) was detected in a small sample of individuals with 22q11.2 deletion syndrome (Andrade *et al.*, 2013), though only one subject presented with generalized epilepsy. Large-scale genetic investigations observed a higher prevalence of 22q11.2 microdeletions in genetic generalized epilepsy (de Kovel *et al.*, 2010; Lal *et al.*, 2015), but specific associations with JME were not substantiated (Helbig *et al.*, 2013), and information regarding concomitant hippocampal abnormalities was not available.

The prevalence figures of HIMAL in our JME and siblings cohort resemble previous observations in patients with a variety of MCDs (Bernasconi *et al.*, 2005). Interestingly, MRI studies detected abnormalities of cortical morphology, surface area and cortical maturation trajectories in JME (Ronan *et al.*, 2012; Lin *et al.*, 2014), while cortical micro-dysgenesis is suggested by neuropathological post-mortem case series (Meencke and Janz, 1984). Hence, we speculate that HIMAL, expressing hippocampal vulnerability to maldevelopment, may represent the common denominator of a spectrum of genetically-underlain developmental disorders associated with epilepsy. A link with a specific epilepsy syndrome, however, seems not to be substantiated.

### Functional implications: neuropsychometry and memory functional MRI

Having identified a spectrum of hippocampal structural abnormalities, we aimed to explore whether patients with JME and their siblings exhibit performance impairment and reorganization of functional MRI activations for functions typically ascribed to the mesiotemporal lobes.

Neuropsychological tests addressing verbal and visuo-spatial learning and recall did not substantiate between-group discrepancies, and there were no differences in recognition memory accuracy on the functional MRI task for both verbal and visual items. Multiple regression models did not identify significant effects of left HIMAL on verbal learning and recall. Collectively, our results do not indicate specific memory deficits in our cohort of people with JME and their siblings. Previous literature addressing memory in JME has provided discordant results (Wandschneider *et al.*, 2012). Pooled estimates from a recent meta-analysis suggest deficits in long-term memory retrieval and storage (Loughman *et al.*, 2014), which were not formally tested in our study. Syndromic heterogeneity may account for the variability of these results. On balance, impaired memory might not represent a prominent trait of JME, as opposed to the more frequently documented dysexecutive features, which may emerge in the context of more prolonged maturational trajectories of executive skills.

Our understanding of the cognitive implications of HIMAL is limited. Our findings, after controlling for potentially confounding variables, do not substantiate an influence of HIMAL on verbal learning and short-term delayed recall scores, in line with reports from a previous paediatric series (Stiers *et al.*, 2010). In the latter study, however, significant performance differences during an executive task were detected for children with epilepsy and HIMAL compared to those without HIMAL, implicating specific dorso-lateral frontal dysfunction. This evidence indicates a complex relationship between hippocampal maldevelopment and cognition, suggesting that potential cognitive implications of HIMAL may be remote from its mesiotemporal site. Future investigations should assess functions subserved by the hippocampus not tested in our study, such as pattern separation, long-term memory retrieval or autobiographical memory, and should aim to identify whether HIMAL may uniquely contribute to executive dysfunction in JME.

Studies providing imaging measures of hippocampal function in JME are scarce. By contrasting brain activation patterns for items remembered with those for forgotten stimuli, subsequent memory functional MRI paradigms specifically address the imaging correlates of successful memory formation (Kim, 2011), and have largely demonstrated the pivotal role of mesiotemporal structures (Squire *et al.*, 2004). Verbal subsequent memory implicates predominant left hippocampal activation (Kim, 2011), as demonstrated here in healthy controls. JME siblings displayed less extensive hippocampal and parahippocampal recruitment, while patients failed to exhibit significant mesiotemporal activation, overall pointing to altered mesiotemporal function in relation to verbal encoding, and implicating phenotypic heritability. Single-subject inspection and group-level parameter estimates for left hippocampal maxima in JME indicated wide confidence intervals, suggesting high heterogeneity. Analysis of extra-temporal activation provides preliminary, uncorrected evidence that profiles of memory-related activation in JME and siblings may also be non-normative for extra-mesiotemporal areas, particularly in frontal locations. With subgroup analyses, we further detected two distinct profiles of dysfunction along the longitudinal hippocampal axis in JME, characterized by anterior mesiotemporal hypoactivation in JME with normal hippocampi, and more posterior activation differences, within the hippocampal body, in JME-HIMAL. As HIMAL is particularly diagnosed based on abnormalities of the hippocampal body, the latter finding may be considered its functional counterpart.

The structure-function analysis revealed a linear relationship between quantitative measures of left hippocampal positioning and activation for successful verbal encoding, independent of hippocampal volumetry. This provides direct evidence that morphological features of the hippocampus may modulate its functional recruitment during a memory task. Of note, normalization to standard space entailed the use of a scanner- and acquisition-specific echo-planar imaging template, derived from a representative sample of healthy controls and epilepsy patients evaluated at our centre, and robust to a spectrum of hippocampal structural abnormalities. Across subjects and in the JME-sibling group, correlation analysis also linked more abnormal measures of positioning with lower recruitment of the middle frontal gyrus, at the same locations where significant activation differences between JME with and without HIMAL were observed. We speculate that this may represent a remote, network-led effect of hippocampal positioning, and may provide a unifying framework to understand the contribution of mesiotemporal abnormalities to the altered frontal lobe function typical of JME (Wandschneider *et al.*, 2012).

In line with previous studies (Bonelli *et al.*, 2010; Sidhu *et al.*, 2013), visual memory activated bilateral anterior hippocampi across all subjects and in the control group. Bilateral mesiotemporal activations were also elicited in JME, though maxima in right parahippocampal gyrus and left posterior hippocampus implicated a non-normative spatial distribution. In JME siblings, visual subsequent memory related to subthreshold mesiotemporal activation. There were no significant differences among the main study groups. Subgroup analyses, however, detected higher left posterior hippocampal activation for visual items in JME-HIMAL, compared with both JME without HIMAL and healthy controls. The latter observations are not paralleled by differences in handedness, language laterality or cognitive performance, and are in sharp contrast with the response typically obtained during visual memory tasks (Sidhu *et al.*, 2013). We therefore propose that this may represent the imaging signature of functional reorganization.

Overall, our findings provide evidence for altered mesiotemporal activation in JME, and to a lesser extent in their siblings, despite normal memory scores, and imply specific effects of hippocampal positioning on mesiotemporal and frontal recruitment in JME with HIMAL. Less extensive abnormalities in siblings suggest lower influence of genetic variables on mesiotemporal functional imaging profiles, compared with structural measures. Across all subjects, a linear relationship is shown between morphological markers of hippocampal positioning and functional recruitment during successful memory encoding. Whether atypical mesiotemporal morphology may affect executive functions and be accompanied by more extensive reshaping of extra-temporal cognitive networks remains subject of future research in JME.

### Limitations

Our study has limitations. Although participants were comparable for estimated intellectual level, and there were no inter-group differences in visual and verbal memory scores, formal matching for education and social status was not attained. The latter aspect is likely not to influence volumetric and morphometric hippocampal assessments, but might represent a confound of our memory functional MRI analyses.

Although none of the participants’ mothers had had a diagnosis of epilepsy, we did not have access to information regarding maternal intake of anti-epileptic drugs (or other drugs/medications) during pregnancy, and we could not establish whether *in utero* exposure to drugs may differ among our study groups. However, we note the absence of documented associations between HIMAL and prenatal drug exposure.

## Conclusion

In patients with JME and their unaffected siblings, morphometric abnormalities of the hippocampus range from subtle hippocampal volume loss to higher occurrence of malrotation and verticalization of the hippocampal body, implicating abnormal shape and positioning. Despite the absence of overt memory impairment, abnormal mesiotemporal recruitment during a memory functional MRI task occurs in siblings and patients, with more marked changes documented for JME-HIMAL. Co-segregation of imaging patterns in both groups is suggestive of genetic imaging phenotypes, independent of disease activity. The hippocampus, and more generally the mesiotemporal lobe, is identified as a neural system affected by genetic variants predisposing to JME.

## Supplementary Material

awz215_Supplementary_Material

## Abbreviations

DITS: dominant inferior-temporal sulcus
HIMAL: hippocampal malrotation
JME: juvenile myoclonic epilepsy
MNI: Montreal Neurological Institute
