# Supplementary material for: Abnormal hippocampal structure and function in juvenile myoclonic epilepsy and unaffected siblings
Source: Brain. 2019 Jul 31;142(9):2670–87. doi: 10.1093/brain/awz215 (PMC6776114; doi:10.1093/brain/awz215)
Supplement: awz215_Supplementary_Material [file awz215_supplementary_material.pdf]

Supplementary online material for:

ABNORMAL HIPPOCAMPAL STRUCTURE AND FUNCTION IN JUVENILE MYOCLONIC EPILEPSY AND UNAFFECTED SIBLINGS

Lorenzo Caciagli[1,2], Britta Wandschneider[1,2], Fenglai Xiao[1,2,3], Christian Vollmar[1,2,4], Maria Centeno [1,2], Sjoerd B. Vos[1,2,5,6], Karin Trimmel[1,2,7], Meneka K. Sidhu[1,2], Pamela J. Thompson[1,2], Gavin P. Winston[1,2,8], John S. Duncan[1,2] and Matthias J. Koepp[1,2]

[1]*Department of Clinical and Experimental Epilepsy, UCL Institute of Neurology, Queen Square, London WC1N 3BG, United Kingdom*

[2]*MRI Unit, Epilepsy Society, Chalfont St Peter, Buckinghamshire, SL9 0RJ, United Kingdom*

[3]*Department of Neurology, West China hospital of Sichuan University, Chengdu, Sichuan, 610041, China*

[4]*Department of Neurology, Ludwig-Maximilians-Universität, Marchioninistrasse 15, 81377 Munich, Germany*

[5]*Wellcome/EPSRC Centre for Interventional and Surgical Sciences, University College London, London, United Kingdom*

[6]*Centre for Medical Image Computing, University College London, London, United Kingdom*

[7]*Department of Neurology, Medical University of Vienna, Vienna, Austria*

[8]*Department of Medicine, Division of Neurology, Queen's University, Kingston, Ontario, Canada*

Correspondence to:

Matthias J Koepp MD, PhD

Professor of Neurology

Department of Clinical and Experimental Epilepsy

Institute of Neurology, University College London

33 Queen Square, London, WC1N 3BG

E-mail: m.koepp@ucl.ac.uk

Running title: Hippocampal abnormalities in JME and siblings

SUPPLEMENTARY MATERIAL 1. Sensitivity analyses for left hippocampal volumetry.

Inspection of Q-Q plots and normality tests revealed normal distribution of hippocampal volumetric data (Shapiro-Wilk test, all p>0.05 for the whole study sample as well for separate analyses in each study subgroup).

For group comparisons of left hippocampal volumes, repeat models using age, gender and handedness produced virtually identical results, and showed convergence of mean hippocampal volume values for patients and siblings [ANCOVA; $F_{(model)}$: 2.7, p=0.027, partial η^2=0.17; $F_{(group)}$=6.01, p=0.004, partial η^2=0.16; *post-hoc* Bonferroni-adjusted p-values: 0.01/0.006, JME/siblings versus controls; p=1.0, JME versus siblings; estimated marginal means (SD), JME/SIB/CTR: 2731mm^3 (249)/ 2668 mm^3 (207)/ 2930 mm^3 (216)].

Inspection of boxplots for left hippocampal volumes identified two outliers above the upper quartile in the JME group, along with one above and one below the lower quartile in the sibling group (shown below, *Boxplot 1*). As sensitivity analysis, we thus repeated group comparisons for left hippocampal volume after excluding outliers. Repeat analyses detected a significant effect of group for left hippocampal volume [JME/SIB/CTR, mean (SD): 2709mm^3 (2709)/ 2666 mm^3 (144)/ 2907 mm^3 (220); one-way ANOVA: $F_{(2,68)}$=8.13, p=0.001, partial η^2=0.20]. *Post-hoc* Bonferroni-corrected tests showed that both JME patients (p=0.002, Cohen's *d*=0.93) and their siblings (p=0.003, Cohen's *d*=1.30) had a smaller left hippocampus than controls. *Post-hoc* comparison of JME and siblings was not statistically significant (p=1.0). Repeat models using age, gender and handedness as covariates again showed virtually identical results [ANCOVA; $F_{(model)}$: 3.94, p=0.004, partial η^2=0.24; $F_{(group)}$=9.14, p=0.0003, partial η^2=0.23; *post-hoc* Bonferroni-adjusted p-values: 0.001/0.002, JME/siblings versus controls; p=1.0, JME versus siblings; estimated marginal means (SD), JME/SIB/CTR: 2698mm^3 (206)/ 2667 mm^3 (204)/ 2928 mm^3 (211)].

These results corroborate findings of the main analysis and show further convergence towards similar values for mean hippocampal volume in patients with JME and their siblings.

Boxplot 1. Left hippocampal volume in JME, unaffected siblings and healthy controls.

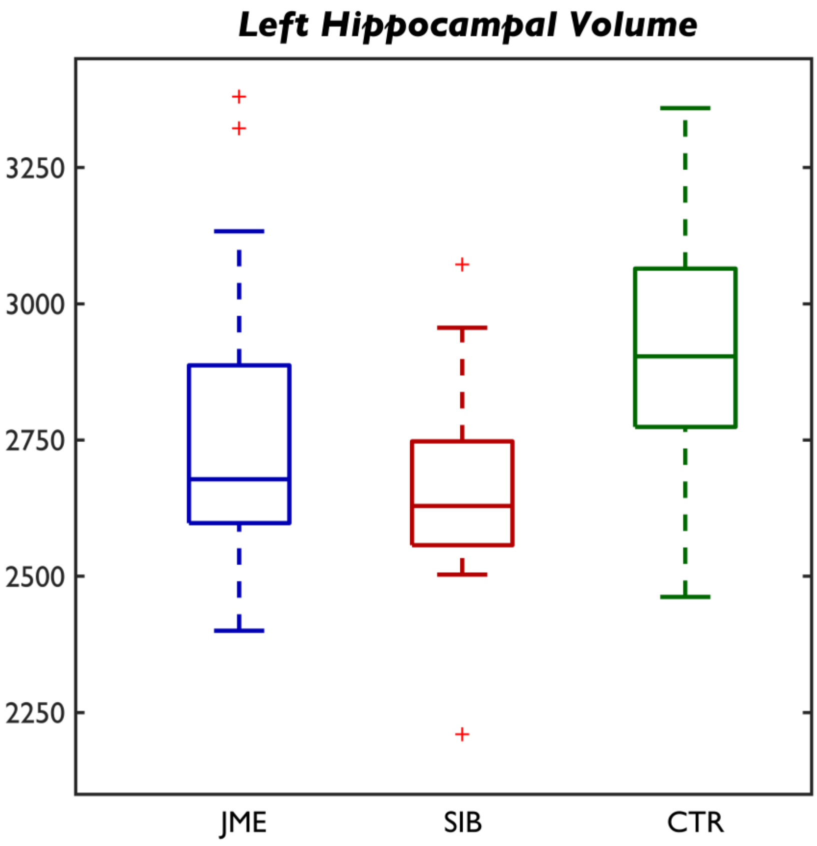

SUPPLEMENTARY MATERIAL 2. Subgroup discrimination via measures of hippocampal volumetry and positioning.

To complement validation of structural hippocampal anomalies in JME and siblings as endophenotypes, we assessed whether quantitative hippocampal measures would be accurate in achieving subgroup discrimination. Using receiver operating characteristic (ROC) curves, we assessed discrimination accuracy of (1) left hippocampal volume and (2) left hippocampal diameter ratio. The latter was chosen because of the significant group effects in the MANOVA for quantitative traits related to HIMAL and the statistically significant *post-hoc* comparisons for JME and siblings versus controls. We also investigated (3) the role of a combination of volume and positioning via entering the above measures in a principal component analysis. The first principal component obtained from left hippocampal volume and diameter ratio (eigenvalue: 1.38, accounting for 69% of the total variance) was considered as a composite left hippocampal marker. We also repeated all the above models controlling for age, gender and handedness. In the latter case, the first principal component

obtained after linear regression of the above covariates from both hippocampal measures had a similar eigenvalue (1.39) and explained an identical proportion of the total variance.

ROC curve analysis identified successful discrimination of patients with JME from controls via left hippocampal volumetric measures and left diameter ratio, with identical accuracy [AUC=0.71, standard error (SE) 0.07, p=0.01 equally in both]. Use of a composite marker improved classification accuracy (AUC=0.74, SE=0.07, p=0.003). Repeat models accounting for covariates showed higher classification accuracy for all measures, with the composite marker (AUC=0.80, SE=0.07, p=0.0002) outperforming individual measures (AUC=0.72/ 0.77, SE=0.07/0.07, p=0.007/0.001 for volume and diameter ratio, respectively).

The above models were also repeated considering JME patients and their siblings as a unitary group. Again, successful group discrimination was achieved for left hippocampal volume and diameter ratio individually (AUC=0.74, SE=0.06, p=0.002 equally in both) and for the left hippocampal composite marker (AUC=0.77, SE=0.07, p=0.0004). Similarly, models accounting for covariates showed higher discrimination abilities for all measures, and the composite marker (AUC=0.81, SE=0.06, p<0.0001) performed better than individual measures (AUC=0.75/ 0.79, SE=0.06/0.06, p=0.001/0.0002 for volume and diameter ratio, respectively).

By showing relatively high discrimination of (a) JME patients and (b) a combined JME-sibling group from controls, this supplementary analysis confirms co-segregation of left hippocampal morphological patterns in patients and their relatives, validating the endophenotypic potential of the quantitative markers identified in our study.

SUPPLEMENTARY FIGURE 1. Representative examples of HIMAL.

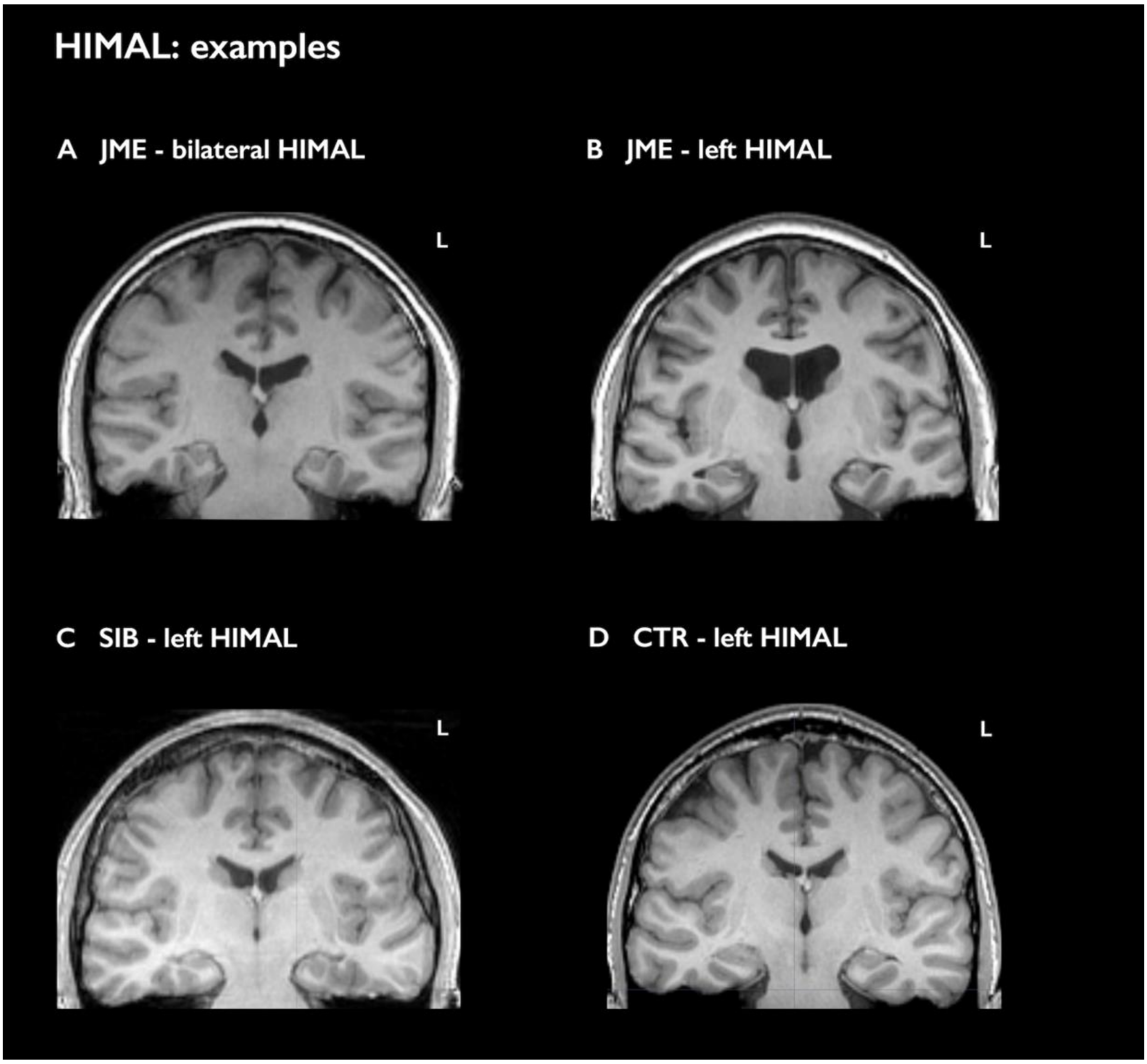

Abbreviations: CTR = healthy controls; HIMAL = hippocampal malrotation; JME = patient with juvenile myoclonic epilepsy; SIB = unaffected sibling of patient with JME. Examples of subjects with JME and bilateral HIMAL (A) or unilateral (left) HIMAL (B) are presented in the first row. In the second row, panel C, left-hand side, a scan of an unaffected JME sibling is shown, where HIMAL is associated with atypical morphometry of the inferior temporal sulci. Panel D provides an example of HIMAL in a healthy control.

SUPPLEMENTARY TABLE 1. Qualitative and quantitative measures associated with HIMAL

Test	HIMAL	Normal	Test Statistic	*P* value	Sensitivity*	Specificity*	AUC
**Shape** (abnormal)	33/35 (94.3%)	2/111 (1.8%)	125.2†	**<0.0001**	94.3%	98.2%	0.98
**Verticalisation of DITS** (verticalized)	25/35 (71.4%)	16/111 (14.4%)	52.5†	**<0.0001**	71.4%	85.6%	0.87
**Lateral hippocampal margin** (loss of convexity)	27/35 (77.1%)	7/111 (6.3%)	81.9†	**<0.0001**	77.1%	93.7%	0.93
**Hippocampal diameter ratio** (%, mean ±SD)	89.6 (12.3)	65.3 (6.7)	230.6^	**<0.0001**	94.3%	95.5%	0.99
**DITS height ratio** (%, mean ±SD)	58.9 (14.8)	25.4 (16.5)	115.6^	**<0.0001**	82.9%	94.6%	0.94
**Parahippocampal angle** (deg., mean ±SD)	98.5 (9.7)	121.7 (9.8)	150.6^	**<0.0001**	97.1%	90.1%	0.98

Abbreviations: AUC= area under the curve; deg. = degree; DITS= dominant inferior temporal sulcus. †Pearson's χ^2; ^*F* statistic, ANOVA. *For quantitative morphological criteria, sensitivity and specificity are reported for cut-off values attaining maximal Youden's index: diameter ratio, 75.4; DITS ratio: 46.1; parahippocampal angle: 110.35.

SUPPLEMENTARY TABLE 2. Verbal subsequent memory – fMRI activation coordinates

Region	*Left Hemisphere*				*Right Hemisphere*			
	**MNI coordinates (x,y,z)**	***Z*-score**	***P* value**	**Parameter estimate (95% CI)**	**MNI coordinates (x,y,z)**	***Z*-score**	***P* value**	**Parameter estimate (95% CI)**
All Subjects								
Anterior hippocampus	**-21 -19 -20**	**2.33**	**0.048**	**0.28 (0.05–0.52)**				
Inferior frontal gyrus	-51 32 10	2.77	0.003	0.34 (0.11–0.57)				
CTR								
Anterior hippocampus	**-18 -10 -20**	**3.23**	**0.009**	**0.27 (0.13–0.42)**				
Anterior hippocampus	**-27 -16 -20**	**3.20**	**0.01**	**0.33 (0.15–0.52)**				
Posterior hippocampus					**27 -37 1**	**3.25**	**0.008**	0.17 (0.08–0.26)
Amygdala	**-21 -1 -14**	**2.92**	**0.019**	**0.17 (0.04–0.30)**				
Parahippocampal gyrus	**-30 -22 -20**	**2.71**	**0.032**	**0.29 (0.13–0.45)**				
Putamen	-21 8 -5	3.17	0.001	0.24 (0.12-0.37)				
Globus pallidum	-27 2 -8	3.13	0.001	0.29 (0.14–0.43)	24 -5 -2	2.83	0.002	0.24 (0.10–0.38)
Middle temporal gyrus	-51 -43 4	4.27	<0.001	0.21 (0.14–0.28)	54 -46 5	3.21	0.001	0.14 (0.07–0.21)
	-69 -28 -8	3.87	<0.001	0.26 (0.16-0.36)				
	-63 58 10	3.48	<0.001	0.34 (0.18–0.48)				
Anterior medial frontal cortex	-2 59 -11	3.50	<0.001	0.66 (0.36-0.96)				
Supplementary motor area					3 20 64	3.49	<0.001	0.38 (0.21–0.55)
Superior frontal gyrus	-15 41 55	3.46	<0.001	0.34 (0.19–0.50)				
	-6 56 31	3.00	0.001	0.47 (0.21–0.73)	3 56 28	2.98	0.001	0.51 (0.23–0.79)
Middle frontal gyrus	-45 17 55	2.89	0.002	0.17 (0.07–0.26)				
Inferior frontal gyrus	-42 23 -8	3.05	0.001	0.45 (0.21–0.70)	51 17 -11	3.23	0.001	0.41 (0.21–0.61)
	-54 35 13	2.93	0.002	0.28 (0.12–0.43)	54 20 -5	3.07	0.001	0.33 (0.16–0.51)
	-51 17 19	2.82	0.002	0.37 (0.15–0.59)				
Insula	-30 -22 1	2.87	0.002	0.17 (0.07–0.26)				
Precentral gyrus					60 2 31	2.84	0.002	0.20 (0.08–0.32)
					36 -16 64	2.50	0.006	0.19 (0.06–0.33)
Paracentral lobule	-3 16 67	3.25	0.001	0.23 (0.12–0.34)				
Supramarginal gyrus	-60 -28 37	2.72	0.003	0.17 (0.06–0.27)				
Angular gyrus	-39 -70 43	2.65	0.004	0.26 (0.09-0.43)				

Precuneus	-6 -61 31	2.64	0.004	0.40 (0.14–0.66)	3 -67 25	3.03	0.001	0.34 (0.16–0.53)
Inferior occipital gyrus	-42 -88 -8	3.05	0.001	0.25 (0.12–0.38)				
JME								
Anterior hippocampus	-21 -19 -20	1.05	0.19	0.16 (-0.27–0.59)				
Inferior frontal gyrus	-48 32 19	1.91	0.03	0.45 (0.01–0.88)				
SIB								
Anterior hippocampus	**-18 -10 -20**	**2.73**	**0.031**	**0.41 (0.14–0.68)**				
Parahippocampal gyrus	**-18 -4 -35**	**2.85**	**0.024**	**0.52 (0.20–0.85)**				
Temporal pole					30 14 -32	2.82	0.002	0.92 (0.41–1.44)
Fusiform gyrus					33 -13 38	2.74	0.003	0.50 (0.21–0.79)
Cerebellum	-24 -64 -23	2.89	0.002	0.49 (0.23–0.75)	36 -49 -29	2.64	0.004	0.55 (0.21–0.89)

Abbreviations: CI = confidence interval; CTR = controls; JME = patients with juvenile myoclonic epilepsy; MNI = Montreal Neurological Institute; SIB = siblings of patients with juvenile myoclonic epilepsy. Coordinates for mesiotemporal and extra-mesiotemporal activations are given in MNI space. When in bold, *P*-values for peak-level mesiotemporal activations are family-wise error rate (FWE) corrected for multiple comparisons using a small volume correction within a 12-mm diameter sphere, centred on the local activation maximum. *P*-values not in bold are uncorrected for multiple comparisons. Parameter estimates (i.e., betas) are reported along with their 95% confidence intervals (CI). For a given anatomical region, statistics are reported for up to three peak-level local activation maxima, ordered by statistical significance. For the JME group, there were no supra-threshold voxels. For completeness, we report coordinates of sub-threshold activation for the two locations showing significant group effects across all subjects (left hippocampus and left inferior frontal gyrus).

SUPPLEMENTARY TABLE 3. Verbal subsequent memory – group comparisons

Region	*Left Hemisphere*			*Right Hemisphere*		
	**MNI coordinates (x,y,z)**	***Z*-score**	***P* value**	**MNI coordinates (x,y,z)**	***Z*-score**	***P* value**
Group effects (F test)						
Amygdala/anterior hippocampus	**-24 -7 -11 (18 -7 17)**	**2.69 (2.60)**	**0.031 (0.033)**			
Middle temporal gyrus	-64 58 7	3.62	<0.001			
	-54 52 13	3.24	<0.001			
Middle frontal gyrus	-33 8 52	3.64	<0.001	45 20 22	2.95	0.002
	-42 4 37	3.37	<0.001			
	-42 8 43	3.10	<0.001			
Inferior frontal gyrus	-48 17 16	2.93	0.002	54 29 1	3.16	0.001
Superior frontal gyrus	-15 50 37	2.81	0.002	21 56 25	2.80	0.003
Cingulate gyrus	-6 -16 31	3.29	<0.001			
Precentral gyrus				27 -19 49	2.86	0.002
Insula	-39 -4 2	3.04	0.001			
Precuneus	-18 -52 40	2.78	0.003	9 -46 31	2.85	0.002
CTR > JME						
Amygdala/anterior hippocampus	**-18 -7 -14**	**2.79 (2.98)**	**0.019 (0.007)**			
Putamen	-30 -10 1	2.50	0.006			
Middle frontal gyrus (anterior)	-45 20 37	2.63	0.004			
Middle frontal gyrus (posterior)	-24 -10 46	3.45	<0.001			
Rolandic operculum				42 -4 19	2.99	0.001
Precentral gyrus				33 -10 40	2.95	0.002
CTR > SIB						
Amygdala/anterior hippocampus	**-24 -7 -11**	**3.08 (2.67)**	**0.009 (0.031)**			
Anterior hippocampus				**30 -10 -17**	**2.39**	**0.049**
Middle temporal gyrus	-63 58 7	4.16	<0.001	66 -46 -1	2.98	0.001
	-54 -52 13	3.80	<0.001			
Putamen	-30 -7 7	3.04	0.001	-30 -4 7	2.97	0.002
Superior frontal gyrus	-15 50 37	3.36	<0.001	9 50 28	3.10	0.001
Middle frontal gyrus	-33 -8 52	4.13	<0.001			
	-42 8 43	3.66	<0.001			
	-39 26 34	3.34	<0.001			
Inferior frontal gyrus	-38 38 -11	3.03	0.001	54 29 1	3.75	<0.001
				45 20 22	3.54	<0.001
Insula	-39 -4 -2	3.92	<0.001			
Precentral gyrus				54 5 34	3.55	<0.001
Anterior cingulate gyrus	-6 -16 31	3.87	<0.001			
Supramarginal gyrus	-57 -22 -40	3.30	<0.001			
Precuneus	-9 -61 -37	3.32	0.001			
CTR > JME-HIMAL						
Hippocampus (body)	**-33 -19 -17**	**2.63**	**0.025**			

		**(2.86)**	**(0.015)**			
Middle frontal gyrus	-39 20 34	3.80	<0.001	42 29 40	3.38	<0.001
	-27 -10 46	3.58	<0.001	27 17 37	2.95	0.002
				33 5 37	3.28	0.001
Superior frontal gyrus	-6 32 43	3.31	<0.001	9 47 42	3.00	0.001
	-18 23 40	3.07	0.001	18 5 49	3.63	<0.001
				18 32 49	3.07	0.001
				21 23 43	3.09	0.001
CTR > JME-noHIMAL						
Amygdala/anterior hippocampus	**-21 -10 -14**	**2.48** (**2.61**)	**0.035** (**0.025**)			
Rolandic operculum				54 -13 13	2.93	0.002
				42 -10 22	2.90	0.002
JME-noHIMAL > JME-HIMAL						
Middle frontal gyrus	-33 23 34	4.14	<0.001	**18 26 34**	**4.45**	**0.031**
	-21 29 28	3.87	<0.001	42 29 40	3.47	<0.001
	-24 44 28	3.32	<0.001	36 26 28	3.43	<0.001
Superior frontal gyrus	-9 35 43	3.68	<0.001	18 20 46	3.87	<0.001
	-18 29 46	3.66	<0.001	21 41 43	3.39	<0.001
	-9 41 49	3.54	<0.001	3 29 52	3.34	<0.001

Abbreviations: CTR= controls; HIMAL= hippocampal malrotation; JME= patients with juvenile myoclonic epilepsy; MNI= Montreal Neurological Institute; noHIMAL= without hippocampal malrotation. SIB= siblings of patients with juvenile myoclonic epilepsy.

Coordinates of mesiotemporal and extra-mesiotemporal group differences are given in MNI space. *P* values for peak-level mesiotemporal activations, all reported in bold font, are family-wise error rate (FWE) corrected for multiple comparisons (small-volume correction) using a 12-mm diameter sphere centred on the local activation maximum. For group differences regarding left mesiotemporal activation, Z-scores and *P* values in brackets refer to repeat models including left hippocampal volume as covariate.

When in bold, *P* values for peak-level extra-mesiotemporal group differences are FWE-corrected for multiple comparisons across the whole brain (e.g. right middle frontal gyrus, JME-noHIMAL vs JME-HIMAL). When not in bold, *P* values for peak-level extra-mesiotemporal differences are uncorrected across the whole brain.

For a given anatomical region, statistics are reported for up to three peak-level local maxima, ordered by statistical significance.

SUPPLEMENTARY TABLE 4. Visual subsequent memory – fMRI activation coordinates

Region	*Left Hemisphere*				*Right Hemisphere*			
	**MNI coordinates (x,y,z)**	***Z*-score**	***P* value**	**Parameter estimate (95% CI)**	**MNI coordinates (x,y,z)**	***Z*-score**	***P* value**	**Parameter estimate (95% CI)**
All Subjects								
Anterior hippocampus	-21 -13 -26	2.48	**0.031**	0.19 (0.06–0.31)				
Posterior hippocampus	-21 -40 4	2.83	**0.013**	0.12 (0.03–0.21)				
Parahippocampal gyrus					18 -25 -17	2.44	**0.034**	0.15 (0.01–0.29)
Fusiform gyrus					42 -46 -26	2.98	0.001	0.14 (0.06–0.24)
Occipital pole					-24 -103 4	2.85	0.002	0.16 (0.05–0.26)
Cerebellum					12 -46 44	2.74	0.003	0.11 (0.03–0.19)
CTR								
Anterior Hippocampus	-21 -13 -23	2.48	**0.04**	0.23 (0.05–0.41)	24 -13 -23	2.56	**0.033**	0.18 (0.05–0.31)
Inferior frontal gyrus					48 35 4	2.48	0.007	0.19 (0.05–0.32)
Occipital pole	-24 -103 4	2.67	0.004	0.27 (0.09-0.46)	27 -100 -8	2.62	0.004	0.24 (0.08–0.39)
JME								
Posterior Hippocampus	-33 -31 -5	2.53	**0.027**	0.16 (0.04–0.29)				
	-21 -40 -4	2.47	**0.031**	0.19 (0.04-0.34)				
Parahippocampal gyrus					18 -28 -14	3.10	**0.006**	0.31 (0.12–0.50)
Anterior cingulate gyrus					3 35 -5	2.83	0.002	0.44 (0.16–0.72)
Fusiform gyrus					42 -46 -29	3.32	<0.001	0.25 (0.12–0.37)
Angular gyrus					57 -67 22	2.80	0.003	0.21 (0.07–0.38)
SIB								
Anterior hippocampus					33 -16 -14	1.72	0.043	0.11 (-0.04–0.27)
Posterior hippocampus	-27 -46 -2	2.32	0.04	0.15 (-0.06–0.36)				
Orbitofrontal cortex	-33 -41 2	2.54	0.006	0.14 (0.04–0.24)				
Superior frontal gyrus	-18 32 61	3.27	<0.001	0.24 (0.13–0.35)				
Fusiform gyrus					33 -61 -2	3.64	<0.001	0.13 (0.08–0.18)
Middle occipital gyrus	-42 -67 -13	2.92	0.002	-0.11 (0.05–0.17)				

Abbreviations: CI = confidence interval; CTR = controls; JME = patients with juvenile myoclonic epilepsy; MNI = Montreal Neurological Institute; SIB = siblings of patients with juvenile myoclonic epilepsy. Coordinates for mesiotemporal and extra-mesiotemporal activations are given in MNI space. When in bold, *P*-values for peak-level mesiotemporal activations are family-wise error rate (FWE) corrected for multiple comparisons using a small volume correction within a 12-mm diameter sphere, centred on the local activation maximum. *P*-values not in bold are uncorrected for multiple comparisons. Parameter estimates (i.e., betas) are reported along with their 95% confidence intervals (CI). For a given anatomical region, statistics are reported for up to three peak-level local maxima, ordered by statistical significance. For the SIB group, there were no supra-threshold mesiotemporal voxels. Coordinates of sub-threshold mesiotemporal activation are reported for completeness.

SUPPLEMENTARY TABLE 5. Visual subsequent memory – subgroup analyses

Region	***Left Hemisphere***			***Right Hemisphere***		
	**MNI coordinates (x,y,z)**	***Z*-score**	***P* value**	**MNI coordinates (x,y,z)**	***Z*-score**	***P* value**
JME-HIMAL > CTR						
Posterior hippocampus	-36 -31 -8	3.08 (2.83)	**0.007 (0.013)**			
JME-noHIMAL > CTR						
Anterior cingulate gyrus				1 35 1	3.80	<0.001
Frontal pole	-42 53 -8	2.75	0.003			
Middle frontal gyrus	-30 -2 46	2.91	0.002	45 26 31	2.67	0.004
Supramarginal gyrus	-36 -46 37	2.77	0.003			
Angular gyrus	-30 -64 22	3.03	0.001	45 -61 19	3.00	0.001
Middle occipital gyrus	-48 82 16	2.95	0.002	30 -91 28	2.61	0.005
	-30 -94 22	2.79	0.003			
JME-HIMAL > JME-noHIMAL						
Posterior hippocampus	-33 -28 -11	2.44 (2.43)	**0.034 (0.036)**			

Abbreviations: CTR= controls; HIMAL= hippocampal malrotation; JME= patients with juvenile myoclonic epilepsy; MNI= Montreal Neurological Institute; noHIMAL= without hippocampal malrotation.

Coordinates of mesiotemporal and extra-mesiotemporal group differences are given in MNI space. *P* values for peak-level mesiotemporal comparisons (reported in bold font) are family-wise error rate (FWE) corrected for multiple comparisons using a 12-mm diameter sphere, centred on the local activation maximum. Z-scores and *P*

values in brackets refer to repeat models including left hippocampal volume as covariate. *P* values for peak-level extra-mesiotemporal differences (all not in bold) are reported as uncorrected across the whole brain.

SUPPLEMENTARY TABLE 6. Structure-function relations – fMRI activation coordinates

Region	***Left Hemisphere***			***Right Hemisphere***		
	**MNI coordinates (x,y,z)**	***Z*-score**	***P* value**	**MNI coordinates (x,y,z)**	***Z*-score**	***P* value**
All subjects						
Hippocampus	-36 -22 -17	3.24	**0.005**			
Middle frontal gyrus	-30 23 37	3.62	<0.001	27 23 31	3.51	<0.001
	-33 17 46	3.57	<0.001	21 26 46	3.45	<0.001
	-24 11 37	3.03	0.001	24 38 43	3.23	0.001
Superior frontal gyrus	-15 47 40	2.93	0.002	24 29 49	3.38	0.001
JME & SIB						
Hippocampus	-36 -22 -17	2.81	**0.015**			
Middle frontal gyrus	-33 23 34	3.49	<0.001	24 23 31	3.35	<0.001
	-33 17 46	3.10	0.001	24 38 43	3.15	<0.001
	-24 29 22	3.08	0.001	33 38 40	3.03	0.001
Superior frontal gyrus	-18 35 37	2.72	0.003	24 29 49	2.96	0.002

Abbreviations: JME= patients with juvenile myoclonic epilepsy; MNI= Montreal Neurological Institute; SIB= siblings of patients with juvenile myoclonic epilepsy. Coordinates for mesiotemporal and extra-mesiotemporal correlational effects are given in MNI space. When in bold, *P*-values for peak-level mesiotemporal activations are family-wise error rate (FWE) corrected for multiple comparisons using a 12-mm diameter sphere (small volume), centred on the local activation maximum. *P*-values not in bold are uncorrected for multiple comparisons. For a given anatomical region, statistics are reported for up to three peak-level local maxima, ordered by statistical significance.
